# The Role of 3D Printing in Planning Complex Medical Procedures and Training of Medical Professionals—Cross-Sectional Multispecialty Review

**DOI:** 10.3390/ijerph19063331

**Published:** 2022-03-11

**Authors:** Jarosław Meyer-Szary, Marlon Souza Luis, Szymon Mikulski, Agastya Patel, Finn Schulz, Dmitry Tretiakow, Justyna Fercho, Kinga Jaguszewska, Mikołaj Frankiewicz, Ewa Pawłowska, Radosław Targoński, Łukasz Szarpak, Katarzyna Dądela, Robert Sabiniewicz, Joanna Kwiatkowska

**Affiliations:** 1 Department of Pediatric Cardiology and Congenital Heart Defects, Faculty of Medicine, Medical University of Gdańsk, 80-210 Gdańsk, Poland; msouzaluis@gumed.edu.pl (M.S.L.); sabini@gumed.edu.pl (R.S.); joannak@gumed.edu.pl (J.K.); 2 First Doctoral School, Medical University of Gdańsk, 80-211 Gdańsk, Poland; agastyap24@gumed.edu.pl; 3 Department of Head and Neck Surgery, Singapore General Hospital, Singapore 169608, Singapore; szymon.mikulski@singhealth.com.sg; 4 Department of General, Endocrine and Transplant Surgery, Faculty of Medicine, Medical University of Gdańsk, 80-214 Gdańsk, Poland; 5 University Clinical Centre in Gdańsk, 80-952 Gdańsk, Poland; finn.schulz@gumed.edu.pl; 6 Department of Otolaryngology, Faculty of Medicine, Medical University of Gdańsk, 80-214 Gdańsk, Poland; d.tret@gumed.edu.pl; 7 Neurosurgery Department, Faculty of Medicine, Medical University of Gdańsk, 80-210 Gdańsk, Poland; jfercho@uck.gda.pl; 8 Department of Gynecology, Obstetrics and Neonatology, Division of Gynecology and Obstetrics, Faculty of Medicine, Medical University of Gdańsk, 80-210 Gdańsk, Poland; jaguszewska.kinga@gmail.com; 9 Department of Urology, Faculty of Medicine, Medical University of Gdańsk, 80-210 Gdańsk, Poland; mfrankiewicz@gumed.edu.pl; 10 Department of Oncology and Radiotherapy, Faculty of Medicine, Medical University of Gdańsk, 80-210 Gdańsk, Poland; ewa.pawlowska@gumed.edu.pl; 11 1st Department of Cardiology, Faculty of Medicine, Medical University of Gdańsk, 80-210 Gdańsk, Poland; rtarg@gumed.edu.pl; 12 Institute of Outcomes Research, Maria Sklodowska-Curie Medical Academy, 03-411 Warsaw, Poland; lukasz.szarpak@bcm.edu; 13 Research Unit, Maria Sklodowska-Curie Bialystok Oncology Center, 15-027 Bialystok, Poland; 14 Henry JN Taub Department of Emergency Medicine, Baylor College of Medicine, Houston, TX 77030, USA; 15 Department of Pediatric Cardiology, University Children’s Hospital, Faculty of Medicine, Jagiellonian University Medical College, 30-663 Krakow, Poland; katarzynadadela@gmail.com

**Keywords:** 3D printing, three-dimensional printing, additive manufacturing, cardiology, surgery, cardiac surgery, urology, obstetrics, gynecology, oncology, radiotherapy, orthopedics, trauma, otolaryngology, head and neck surgery, mandible reconstruction, training, simulation, outcomes

## Abstract

Medicine is a rapidly-evolving discipline, with progress picking up pace with each passing decade. This constant evolution results in the introduction of new tools and methods, which in turn occasionally leads to paradigm shifts across the affected medical fields. The following review attempts to showcase how 3D printing has begun to reshape and improve processes across various medical specialties and where it has the potential to make a significant impact. The current state-of-the-art, as well as real-life clinical applications of 3D printing, are reflected in the perspectives of specialists practicing in the selected disciplines, with a focus on pre-procedural planning, simulation (rehearsal) of non-routine procedures, and on medical education and training. A review of the latest multidisciplinary literature on the subject offers a general summary of the advances enabled by 3D printing. Numerous advantages and applications were found, such as gaining better insight into patient-specific anatomy, better pre-operative planning, mock simulated surgeries, simulation-based training and education, development of surgical guides and other tools, patient-specific implants, bioprinted organs or structures, and counseling of patients. It was evident that pre-procedural planning and rehearsing of unusual or difficult procedures and training of medical professionals in these procedures are extremely useful and transformative.

## 1. Introduction

**Figure 1:**
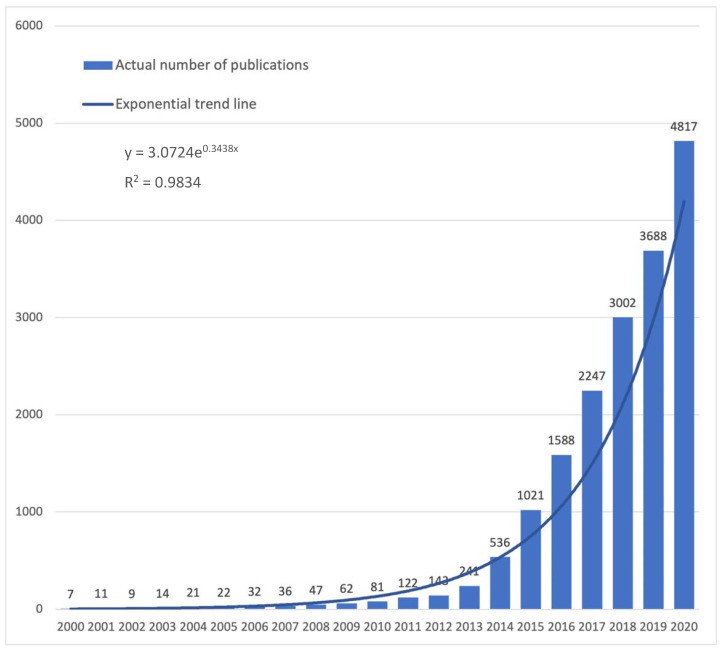
The number of PubMed indexed publications since 2000 (total 19,562) and fitting exponential trend line. Search query: 3D printing or additive manufacturing.

Three-dimensional printing (3D printing, 3DP), also known as rapid prototyping or additive manufacturing, is not new in the tech industry. In fact, the first patents and prototypes in this field date back to the 1970s, with early additive manufacturing equipment and materials following in the subsequent decade. Various manufacturing methods have been developed, such as fused deposition modeling (FDM), selective laser sintering (SLS), stereolithography (SLA), to name a few. However, a major breakthrough in the broad-scale implementation of this technology happed after 2010, following the expiration of many relevant patents. This enabled the development of affordable desktop 3D printers, which further democratized 3DP technology and made it more accessible to the public [1]. This shift, in turn, led to the exploration of various medical implementations. While there were singular mentions of 3DP technology in PubMed prior to the year 2000, the subsequent two decades have shown exponential growth in the number of publications (Figure 1), a trend suggestive of a disruptive potential of 3DP technology.

### 1.1. Three-Dimensional Printing Technique Overview

**Figure 2:**
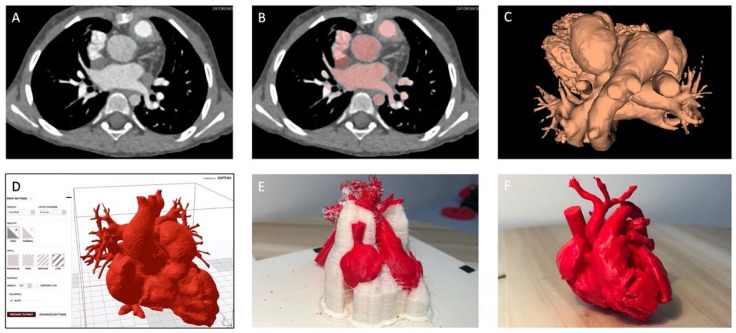
The steps in the process of 3D printing. (**A**). Source CT; (**B**). CT after segmentation (red); (**C**). Digital model (STL); (**D**). Print setup; (**E**). Raw print covered in support material; (**F**). Final 3D printed model. Detailed descriptions in text. (If not stated otherwise, images courtesy of Jarosław Meyer-Szary, Department of Pediatric Cardiology and Congenital Heart Defects, Faculty of Medicine, Medical University of Gdańsk, Gdańsk, Poland).

**Figure 3:**
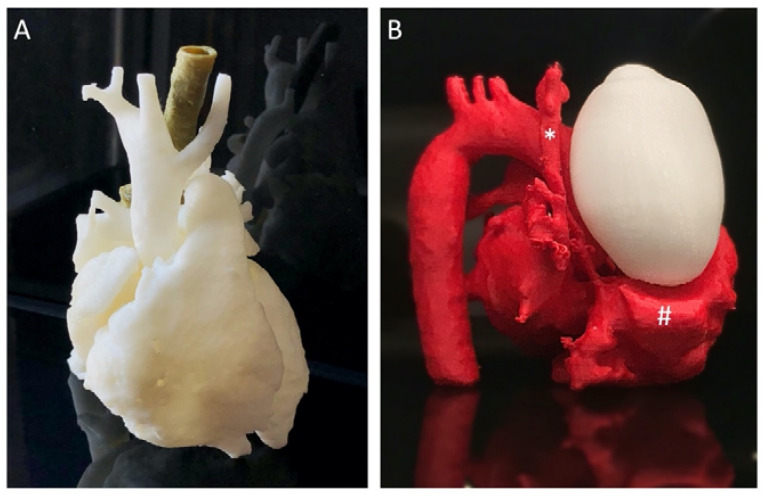
Examples of multi-part models. (**A**) a case of aortic ring, to visualize a compressed trachea by adjacent, abnormally positioned vessels, a cardiovascular model is printed in white and, separately segmented, trachea is printed green and fitted in place; (**B**) a case of neonate with a mediastinal tumor (white) compressing the cardiovascular structures (red), the superior vena cava (asterisk) and the right atrium (hashtag), the model was based on multimodality imaging owing to the fact that Angio CT has superior special and temporal resolution for cardiovascular structures while MRI has superior ability to differentiate soft tissue structures and tumors. Parts of the models coming from different modalities were assembled in post-production.

Patient-specific 3D models of anatomical structures can be generated from various medical imaging modalities in a multi-step process we have detailed previously [2]. Most commonly, computed tomography (CT) and magnetic resonance imaging (MRI) volumetric datasets are used (Figure 2A). Transesophageal echocardiography (TEE) rotational angiography can also provide high-quality source image data. Irrespective of the modality employed, superb image fidelity is essential for quality 3D printing, as imaging faults and artifacts will complicate pre-print processing and, in the worst-case scenario, may be carried over to the final print [2,3,4]. On special occasions, multimodality imaging is employed to leverage the strengths of more than one imaging modality (Figure 3).

The source images are imported from Digital Imaging and Communications in Medicine (DICOM) format into a specialized software platform designed for medical image analysis and processing (Figure 2A). Segmentation (Figure 2B) is the key step of identifying target anatomical elements and excluding extraneous components through an automated, manual, or semi-automated process. This segmented dataset is converted into a STereoLithography (STL) file (Figure 2C) and passed on to printer-specific software to set up print parameters (Figure 2D). The resultant file is passed on to the printer. Numerous 3D printing techniques and a vast array of materials are available, with unique properties such as the type of the print process, print time, model durability, compliance, flexibility, and translucency, which lend themselves to various applications. Once the printer is set up and the print is started, little to no user intervention is required. However, the newly-printed model is rarely finished at this point (Figure 2E). Typically, post-processing is required before a finished model (Figure 2F) is made and ready to be used in, for instance, a procedure simulation. This post-processing may include removal of support parts (manually or by dissolving), sanding and polishing, chemical smoothing, UV curing, assembly in case of multi-part models (Figure 3), etc. Each of the steps (image pre-processing and digital model creation, actual printing, and print post-processing) are relatively time-consuming, taking a number of hours on average, while most (with the exception of printing itself) also tend to be labor-intensive. Overall, this currently renders the 3DP process sub-optimally suited to urgent applications or emergency situations.

### 1.2. Application of 3D Printing in Medicine

**Figure 4:**
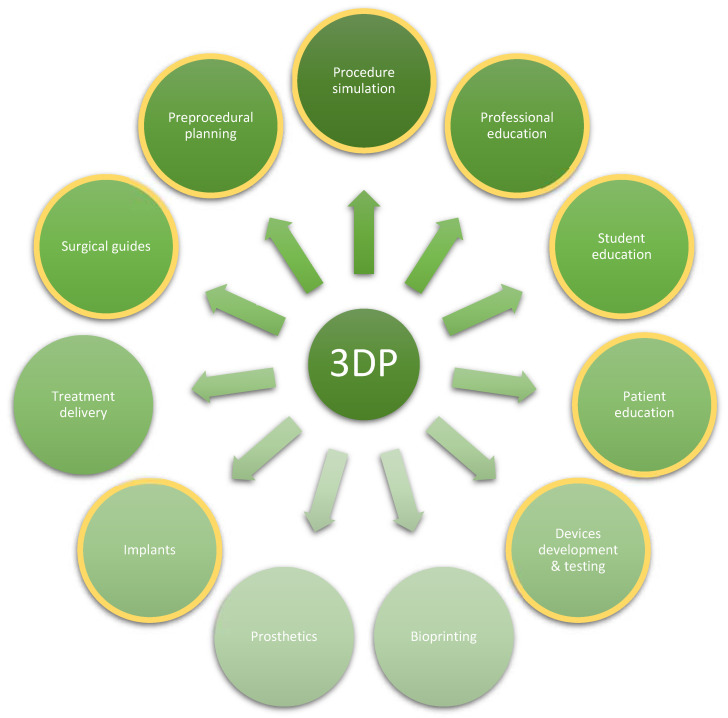
Breadth of medical applications of 3D printing. Highlighted in yellow are those in focus in this review. The list is non-exhaustive, and new applications are evolving.

Despite the limitations, 3D printing has found its way to multiple applications in various fields of medicine, as depicted in Figure 4, with each field having come a long way over the recent decades. Overall, the chief advantage of 3DP is the ability to transform two-dimensional imaging of a patient, once merely viewable on a flat screen, into a tactile 3D model that can be appreciated in real space, handled, physically manipulated, and even deformed. Clinical applications, which deal with complex anatomical relationships and rely on advanced imaging modalities to make judgments and plan possible interventions, are poised to derive the greatest benefit from advances in 3DP.

Likewise, improvements in 3DP technology have created new avenues for the development and applications across other specialties and domains. Today, 3D printing is being progressively integrated into and has begun to reshape surgical practice as well as surgical training, with recent reports having shown possible uses of 3DP across several subspecialties. Perhaps best suited to take advantage of 3DP are those that deal with the skeleton. The significantly higher density of bone compared to surrounding soft tissue facilitates imaging segmentation and thus lends itself optimally to 3D modeling. However, specialists in multiple fields, whether dealing primarily with bone or with soft tissues, are poised to derive benefits from 3DP technology as an adjunct to pre-procedural planning in education, training, simulation, or practice ahead of complex, uncommon or high-risk procedures, or as means of surgical guidance, or even for custom 3D-printed tools, devices, and patient-specific prostheses and implants.

Selected examples of the various applications of 3DP are showcased in the following sections, as pertaining to the respective individual disciplines of pediatric (cardiac) surgery, and interventional cardiology, adult general and orthopedic surgery, otorhinolaryngology, head and neck surgery, neurosurgery, gynecology, and obstetrics, urology, as well as emergency medicine, anesthesiology, and radiation oncology.

From education, through diagnostics, to definitive treatment, the modern multidisciplinary approach has progressed to a stage where individualized therapy has become simpler and more broadly accessible. This is further facilitated by the implementation of 3DP technology with models being utilized for surgical planning, simulation-based training and education, developing surgical guides and other tools, as well as counseling of patients. Manufacturing options range from Fused Deposition Manufacturing (FDM) Polylactic Acid (PLA) prints of fracture sites, used for pre-operative planning, to more complex Direct Metal Laser Sintering (DMLS) Titanium prints of patient-specific implants, and finally, bio-implants 3D printed with bio-ink currently undergoing animal testing. Current clinical applications, shown to benefit directly from the involvement of 3DP models, ranging from treatment of pectus excavatum, diaphragmatic hernia, hydrocephalus, tracheobronchomalacia, as well as oncologic resection and reconstruction, to name a few. Hence, 3D printing clearly has a wide area of potential applicability (Figure 4). However, it is not yet established as the first method of choice, with traditional solutions preferred in many centers. Nevertheless, moving forward, 3D printing offers extensive opportunities not only to improve treatment and patient outcomes but also to enable advanced training of future physicians and surgeons. It can also be used as a valuable tool for patient education prior to complex surgical procedures or less invasive interventions.

The aim of the following article is to provide the reader with an introduction, broad cross-sectional overview, and assessment of 3D printing across various medical fields. The current state-of-the-art, as well as real-life clinical applications of 3D printing, are reflected in the perspectives of specialists practicing in the selected disciplines, with a focus on pre-procedural planning, simulation (rehearsal) of non-routine procedures, and on medical education and training.

## 2. Discipline Specific Applications

### 2.1. Pediatric Cardiac Surgery

**Figure 5:**
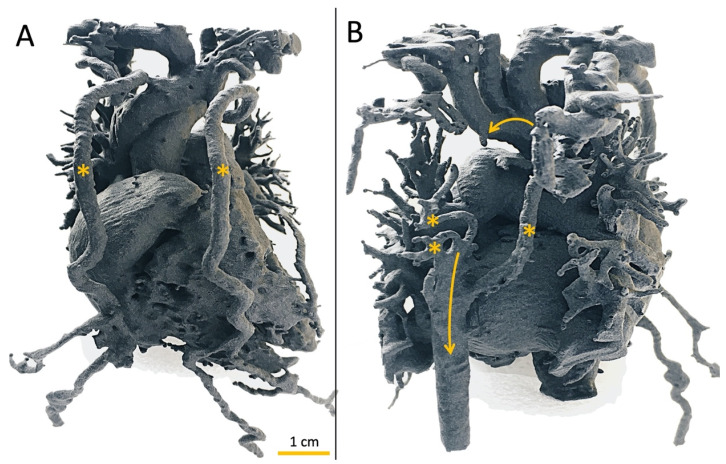
Cardiovascular model of an interrupted aortic arch of a 3.5 kg neonate made to facilitate preoperative planning. SLS method was chosen for its superior handling of tiny details especially given no supports are necessary for the process. (**A**) Anterior view, dilated mammary arteries as part of collateral circulation (asterisk). (**B**) Posterior view, other collateral arteries (asterisk); top (curved) arrow—aortic arch; bottom (straight) arrow—distal part of a thoracic aorta; 2.5 cm gap between the arrows is where a surgeon needs to place anastomosis.

Pediatric cardiac surgery mostly deals with congenital heart diseases. It is not uncommon to deal with cases of very unusual anatomy, and considering those are often neonates and infants with tiny hearts and vessels (Figure 5A,B), a well-prepared surgical plan is a key to the treatment success. The advent of 3DP allowed making the best use possible from imaging modalities such as computed tomography (CT), cardiac magnetic resonance imaging (MRI), or three-dimensional echocardiography to visualize cardiovascular structures and their surroundings better. Currently, 3DP allows not only to study specific patients’ anatomy but to actually attempt treatment and choose the most promising approach.

Scanlan et al. created models of pediatric atrioventricular valves for physicians and pediatric cardiac fellows to simulate two different types of valvular repair. They used transthoracic echocardiographic images as a source. They compared two production methods—PolyJet printing with TANGOPLUSFLX 930 (Shore A ~27) and silicone molding. Both types were accurate in terms of anatomical shape and dimensions, but silicone models were found to be superior in terms of visual realism (*p* = 0.04), suturing feel (*p* = 0.01), and mimicking of repair (annuloplasty *p* = 0.02, CAVC repair *p* = 0.03) [5].

In a multicenter study, Valverde et al. judged the effects of using 3DP models in the course of treatment of congenital heart defects. Ten hospitals around the world selected 40 pediatric patients (mean 3 years, ranging from 1-month-old to 34 years old) to print models to understand the individual’s pathology better. Most surgeons (96%) felt that having this medium to visualize the pathology helped to improve outcomes. In 47.5% (CI: 29.6–61.5%) of cases, 3DP models resulted in a change in surgical decision, 10 of which were significant. Two cases were able to be reassessed, initially planned for biventricular repair; in the end, the preferred method of univentricular heart staged palliation was decided upon as the 3D model was able to demonstrate that the ventricular septal defect (VSD) was not adequate for septation hence reducing possible unforeseen complications [6].

Deng et al., in an RCT study, used personalized patient 3DP models with VSD to convey information about the pathology and the planned surgery to patient guardians (*n* = 20). Several factors were found to be improved when compared to the control group. A better understanding of the ventricular septal defect (*p* = 0.02) as well as a better understanding of the intricacies of the procedure and possible complications (*p* = 0.02) [7].

Costello et al. used 3DP models in simulation-based training to help residents better understand the subtypes of VSD and the management process from diagnosis to intervention. They found that when compared to their knowledge before the seminars, they had significantly increased their knowledge acquisition (*p* < 0.001), knowledge reporting (*p* = 0.01), and structural conceptualization (*p* < 0.001) [8].

In patients with unclear treatment options who had complicated congenital heart defects, Tiwari et al. used 3DP models to get a better grasp on the pathology and determine the best route of intervention. Ten patients were selected to establish the best path to move forward. For eight patients, the treatment decision changed after seeing the 3DP model, and all patients had no postoperative complications [9].

Those are a few examples of the use of 3DP in the field of pediatric cardiac surgery. They show not only what is possible but also what are the benefits of this new approach to planning such surgeries. Limiting anesthesia time, overall surgery time, bleeding, X-ray exposure, etc., while improving the accuracy of the procedure may be crucial to the long-term outcomes of those youngest patients.

### 2.2. Pediatric Interventional Cardiology

Pediatric interventional cardiology is oftentimes a less invasive alternative to treating congenital heart diseases. Without cutting the patient open medical instruments and implants are introduced to the patient’s heart via peripheral veins or arteries. Treatment of more and more complex or unusual cases is attempted, and 3DP offers the opportunity to plan and simulate such non-standard treatments. 

In order to determine how a 3DP can be useful in planning endovascular stenting in transverse aortic arch hypoplasia in a 15-year-old boy, Valverde et al. created two models (rigid and flexible) of an aorta with a hypoplastic aortic arch based on an MRI study. They then used this model to simulate the surgical therapy and compared the results of the simulation to that of the true intervention. Based on a series of measurements at eight different anatomical sites, the model was found to be an excellent match to ground truth, only slightly overestimating the results (0.36 ± 0.45 mm on average). Additionally, the assessment of optimal stent position, size, and length was found to be useful in procedure planning [10].

Phillips et al. used patient-specific 3DP models of patients with tetralogy of Fallot who already had transannular patch intervention that then required transcatheter pulmonary valve replacement. This was performed to help create patient-specific strategies to carry out this complex procedure that would normally not be candidates for the procedure. Valve implantation was successful in all patients (*n* = 8), and one patient had a minor complication. The use of this methodology was deemed effective to give these unique patients more treatment strategies [11].

An excellent example of the potential of 3D printing in preprocedural planning in non-standard situations is described by a Canadian team of cardiologists. They carried out hybrid melody valve implantation in mitral position in an infant born with an atrioventricular septal defect that previously underwent three surgical valve reconstructions with no good effect. Prior to the procedure, there was substantial apprehension that the implanted valve would obstruct the left ventricular outflow tract (LVOT), considering low weight, unusual anatomy, and a non-standard treatment plan. The custom-made model allowed for the simulated insertion of the valve and in vitro assessment of proper anchorage of the valve to ensure no LVOT obstruction would result from the procedure. The treatment was carried out to plan with excellent clinical results [12].

Bhatla et al. elected to create a 3DP model of one of their patients in order to understand that patient’s complex anatomy better. Being able to handle a model of the patient’s heart allowed them to select an unorthodox viable method to close a ventricular septal defect by anticipating the need for a right atriotomy with a tricuspid valve approach [13].

The opportunity to plan and rehearse a treatment approach never attempted before gives the physician confidence and opens new possibilities for the patient leading to better outcomes.

### 2.3. Cardiac Structural Interventions

**Figure 6:**
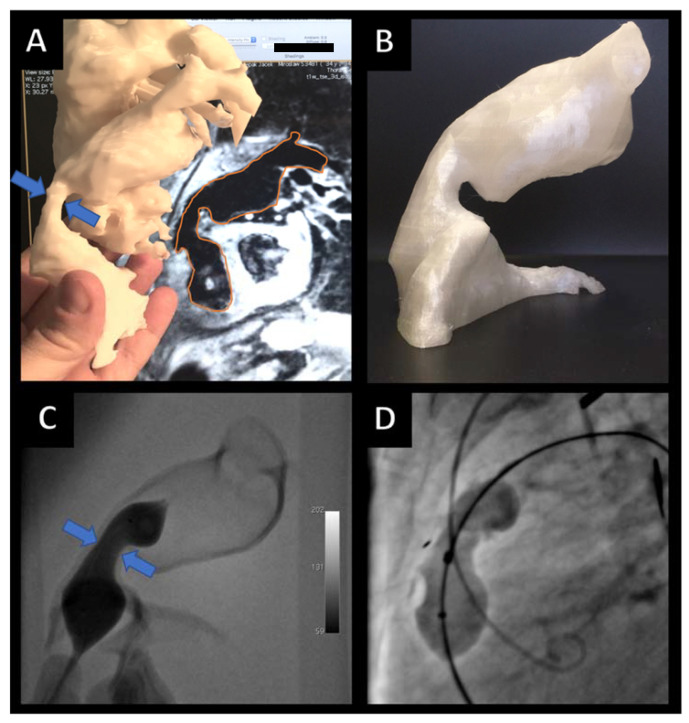
A set of two MRI-based models for planning and simulation of cardiovascular intervention—implantation of a Melody valve across a secondary pulmonary artery conduit stenosis (arrows) in an adult born with tetralogy of Fallot previously treated at the age of six by surgical implantation of pulmonary artery conduit, which has now become near-obstructed (arrows). (**A**) 3D-printed blood pool anatomical model including the pulmonary artery and aorta. (**B**) 3D-printed hollow model. (**C**) Fluoroscopic image of contrast-filled angioplasty balloon catheter across the stenosis. (**D**) Pre-dilatation fluoroscopic image of the implantation site (landing zone) using a similar balloon catheter during the actual intervention. The case was previously discussed in detail elsewhere [14].

Structural heart disease interventions are a new and quickly-expanding discipline with novel devices, implants, techniques, and strategies at hand as well as on the horizon (Figure 6). This evolution opens new opportunities but also adds to the complexity of already challenging procedures. Specifically, one important application is in the treatment of pediatric cardiac patients. This is because the age (and size) of the child influences the selection of the method of intervention. Moreover, the smaller the patient, the more complex the procedures tend to be, which renders 3D modeling especially useful in this patient population.

Transcatheter Pulmonary Valve Replacement (TPVR, also known as Percutaneous pulmonary valve implantation, PPVI) is a minimally invasive alternative to surgical treatment for patients with pulmonary stenosis or insufficiency. A majority qualified are adults with congenital heart disease (ACHD, also known as grown-up congenital hearts, GUCH) patients once treated for tetralogy of Fallot or similar pathologies [15]. The variable anatomy of a right ventricular outflow tract [14,16], the landing zone, may be the greatest challenge. With 3D-printed models (Figure 6A,B), it is possible to inspect the regional anatomy better, simulate the procedure in a specific patient (Figure 6C), and carry out the implantation (Figure 6D) to an excellent outcome [14].

Ripley et al. conducted a retrospective study using patients that underwent transcatheter aortic valve replacement to see how accurate these models react to the intervention when compared to the procedure. Pre-operative CTs were used (*n* = 16) and found to be highly accurate in anatomy (with a 0.1 mm tolerance to the diameter) and were able to correctly predict paravalvular aortic regurgitation (6 out of 9) and non-paravalvular aortic regurgitation (5 out of 7, *p* = 0.13 by Chi-square) [17].

Zelis et al. wanted to compare custom 3DP silicon valvular models to actual patient pressure gradients pre- and post-transcatheter aortic valve implantation (TAVI) when placed in a model of a circulatory system. After measuring the replicated pre- and post-TAVI pressure gradients, they found that the measurement prior to the intervention was underrepresented in the 3DP model (35.1 ± 0.6 mmHg as compared to 45.3 ± 1.5 mmHg) but resembled the patient gradient in the post-intervention measurement (3.7 ± 0.7 mmHg as compared to 6.7 ± 2.3 mmHg) [18].

In order to develop a system for preoperative planning in TAVI, Qian et al. conducted a retrospective study and created patient-specific 3DP models for 18 patients and tried to determine how accurate these models were to predict negative outcomes. It was found that they were able to accurately predict paravalvular leaks in 75% of the patients (*n* = 9) and in moderate-to-severe paravalvular leaks annular calcification (95% CI: 58%–96%, *p* < 0.001) and bulge index (95% CI: 51%–93%, *p* = 0.04) were accurate predictors [19].

**Figure 7:**
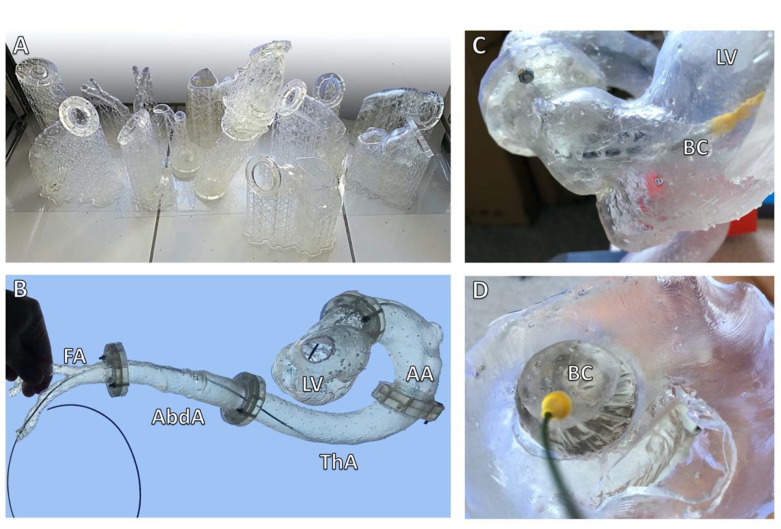
Three-dimensional-printed model for transcatheter aortic valve implantation (TAVI) procedure training. (**A**) Parts for two different models at the post-processing stage—UV cured but waiting for support removal and assembly. (**B**) Assembled model with a catheter inserted. (**C**) Closeup of the left ventricle showing a balloon catheter inside. (**D**) View from the inside with the balloon catheter inflated. FA—femoral arteries, AbdA—abdominal aorta, ThA—thoracic aorta, AA—aortic arch, LV—left ventricle, BC—balloon catheter.

These models can be utilized for personnel training for the TAVI procedure as well as any other vascular or valvular intervention. By using imagery from previously treated patients, it is possible to make a whole series of models realistically representing actual anatomy (Figure 7). This makes 3DP superior to stock simulators that usually represent idealized anatomy and are available with little variety.

A distinct and still growing subset of patients is those with structural valve deteriorations (SVD) of previously implanted, either surgically or percutaneously, valves. These so-called valves in valve procedures are associated with an increased risk of serious complications such as coronary obstruction, valve under expansion, and thrombosis. Given the complex potential interplay between the old and new valves rings/scaffolds, as well as the degree of failed bio-prosthesis leaflet displacement, those life-threatening complications are hard to predict using conventional means. Benchtop transcatheter heart valve (THV) deployment in 3DP models helps assess those risks and facilitate procedure planning, prompting the decision about the need for aortic scallops laceration (i.e., BASILICA procedure) [20].

Another rapidly evolving field is percutaneous mitral interventions, where due to the greater complexity of valve apparatus, preprocedural planning is of great importance [21]. Little et al. showed that 3D-printed models reliably represent the size and anatomy of mitral leaflets, facilitating the successful positioning of MitraClip (Abbott Laboratories, Abbott Park, Illinois). The use of the models was suggested to reduce in vivo movement of the posterior leaflet and the degree of mitral regurgitation. In patients considered for the transcatheter mitral valve replacement procedure (TMVR), 3D-printed models can predict left ventricular outflow tract obstruction, indicating the need for anterior mitral leaflet laceration (LAMPOON procedure) or septal ablation as a preventive measure [22]. Mitral valves models printed from materials of different elastic properties and using the pump mimicking blood flow enabled simulating percutaneous techniques in the in vivo-like conditions, allowing for proper sizing of the annulus for TMVR and its positioning [23].

Percutaneous left atrial appendage (LAA) closure is no longer an experimental novelty but a guideline-recommended treatment of choice for high-risk patients for stroke with atrial fibrillation and contraindications for anticoagulation [24,25]. The successful procedure depends on multiple variables such as recognizing the anatomical type of the LAA, device choice, and sizing. In this procedure, 3D printing proved useful in improving the operator’s learning curve [26], identification of the optimal transseptal puncture site, and device sizing [26,27,28,29]. In left atrial appendage occlusion, Li et al. compared two groups, one simulating the procedure on a 3DP model (*n* = 21) and the control using normal methods (*n* = 21). Three mild residual shunts were found in the postprocedural transesophageal echocardiography of the control group compared to none in the 3DP group. A statistically significant difference was also found in radiation exposure (561.4 ± 25.3 mGy vs. 651.6 ± 32.1 mGy, <0.05) [30].

Meyer-Szary et al. used 3D-printed models to plan an unusual procedure to close an aneurysm located at the lesser curvature of the aortic arch. The models helped to choose optimal vascular access as well as proper closure device [4]. The postoperative follow-up showed a successful outcome [31].

A similar approach was also successfully used for preprocedural planning of transcatheter paravalvular leak closure (PVL) with AVP III plugs (Abbott Laboratories, Abbott Park, Illinois) by Cruz-Gonzalez et al. [32]. More recently, ElGuindy et al. published a case series of large, complex PVL occlusions preceded by bench testing on 3D-printed models. Dimensions of paravalvular leaks mandated using multiple occlude to seal the regurgitant orifice. Before attempting the therapeutic procedure, different plug configurations were tried on a printed model to achieve the best sealing with no interaction between the plugs and the valve [33].

3D-printed models and bench-testing provide the operator with invaluable information of the type, size, and the number of devices needed and help to avoid interference with mechanical valve disc in this demanding subset of percutaneous interventions.

### 2.4. Pediatric Surgery

All pediatric surgeries tend to be complicated by the range of sizes of each patient, a tiny infant to a grown teenager. In order to improve the surgical interventions, new custom devices can be better suited for the individual patient and help improve outcomes. Being able to apply therapies to models helps improve outcomes. New opportunities to create custom patient-specific models help tailor procedures to the patient. 

Bellia-Munzon et al. used 3DP models to aid all steps in the minimally invasive approach for pectus excavatum (PE) Nuss repair. In a 4-year timespan, personalized templates and implant tools were created for 130 patients. Optimal prints with anatomic match were created in 92.3% (*n* = 120); implant rebending was only needed in 5.4% of the patients. They too found a decrease in operation time (87.6 ± 49.9 min compared to 125.4 ± 30.7 min, *p* < 0.0001) as well as fewer bars used (2.6 ± 0.5 versus 1.7 ± 0.6, *p* < 0.001) [34].

Huang et al. showed that by creating 3D models of patients with pectus excavatum, they could help surgeons improve outcomes for the Nuss procedure. When comparing the use of 3DP models for preoperative preparation (*n* = 15) to the traditional Nuss procedure (*n* = 342) it showed a decrease in operation time (60.36 ± 20.6 min versus 74.34 ± 18.0 min, respectively, *p* < 0.001) and fewer bars used (1.00 ± 0.00 versus 1.36 ± 0.48, respectively, *p* < 0.001) [35].

With the use of 3D-printed vacuum bells from the patient’s chest surface scans, Deng et al. created patient-specific therapies for correcting pectus excavatum. Used as a non-surgical alternative to Nuss surgery, they were able to show that after an average of 11.1 months of therapy for 30 min 1.5 times a day, patients experienced significant improvement (z = −4.569, *p* < 0.001, 30 patients analyzed). Longer use showed better outcomes (R^2^ = 0.235, *p* = 0.014) [36].

In order to leverage the advantages of using 3DP models for preoperative planning, Villarreal et al. first conducted a literature review for conjoined twin separation assisted by 3D models. The procedure is inherently complex, and the 10 case reports (17 procedures) found that it was an indispensable tool leading to 14 successful surgeries with minimal complications, with the remaining resulting in a single twin death or some complication unrelated to the separation [37].

Prayer et al. used in vivo MRI scans for the planning and qualification of patch placement in congenital diaphragmatic hernias. It was found that calculated defect–diaphragmatic ratios were significant (*p* < 0.001), and the 3DP helped predict patch placement [38].

Sánchez-Sánchez et al. aimed to improve patient outcomes by trying to decrease surgical complications in complex oncological patients. By using their high-resolution MRI and CT scans to 3D print models, the oncological surgeons were able to use the models as references to accomplish their surgeries in four pediatric patients without incident. Case 1: removal of a Wilms tumor affecting both kidneys; case 2 three Wilms tumor metastatic lesions in the lungs; case 3: poorly differentiated neuroblastoma in the abdomen; case 4: left paravertebral ganglioneuroma affecting the trachea, the internal jugular vein, the carotid artery, and the vertebral artery [39]. 

Beltrami et al. studied the use of 3DP custom-made implants in 11 pediatric patients with skeletal cancers that required resecting the bone. The overall Musculoskeletal Tumor Society Score of either good or excellent in 87.5% of patients. The custom implants seemed to yield benefits, but some patients were lost due to the progression of the disease, and in one case, removal of the implant was necessary due to infection. However, the results were promising [40].

Vanesa et al. treated mandibular hypoplasia using 3DP models to plan and custom 3DP implants to conduct mandibular distraction in five patients. Surgical planning was subjectively found to be easier, and the final results of the treatment had only minor issues with results comparable or better than other reported studies without the aid of 3DP planning and implants [41].

Materials that can change over time are especially useful in the growing bodies of pediatric patients. Designing and printing airway splints for tracheobronchomalacia that meet the need of the specific pathology are being developed by Morrison et al. Bioresorbable splints for three patients matched their need by growing with the individual as no difference in airway caliber measurements at 1-month postoperatively (for all three patients, *p* < 0.01) to 30 months (for one patient, as the other two were still being followed up on, *p* < 0.01) between the treated airway and the normal side [42]. Showing that it is a feasible process is a great example of how this method can be applied to in-hospital medical manufacturing, the future of personalized medicine.

Using 3D prints as an aid for procedures has the potential to enhance the quality of surgical procedures. The range of sizes of pediatric patients leads to gaps in medical equipment that can ideally serve certain individuals who fall between the readily available tools. Supplementing these gaps with custom prints has helped raise the quality of interventions.

### 2.5. General Surgery

In relation to abdominal surgery, 3D-printed anatomical models are most frequently created for kidneys and the liver [43]. These models are usually generated to assess resectability of tumor mass, plan living donor transplantation, plan approach for percutaneous kidney stone removal, and develop models of an extrahepatic biliary tree for training endoscopic and laparoscopic procedures [44,45,46,47,48]. Additionally, several reports have described the use of 3D-printed models of other abdominal structures such as the pancreas, spleen, and abdominal vessels [49,50,51,52].

In order to treat 10 patients with pancreatic cancer or cancer around the ampulla of Vater, Fang et al. use 3DP models to help with their surgical planning. All surgeries resulted in R0 margins with operation times of 347 ± 36 min, interoperative blood transfusions of only 250 ± 196 mL, with minimal postoperative hospitalization time of 12.0 ± 2.7 days [52].

Bauermeister et al. were able to find, in their review, that 3DP skull models enabled a surgical team to critically visualize structures and their spatial relationship with surrounding anatomy, enhancing their confidence as well as reducing complications. Three-dimensional printing also provides an effective and cheap process to create patient-specific, customized implants such as interphalangeal, vertebrae, and mandibular joints [53].

In order to better understand the viability of 3DP models in assisting laryngotracheal stenosis surgical correction, Richard et al. considered two patients, one with grade 4 subglottic stenosis and another with grade 4 tracheal stenosis. They found that with the aid of computational fluid dynamics, the 3DP model could be used to evaluate the benefit of the surgical intervention [54].

Erdogan et al. compared personalized 3DP external nasal splints to thermoplastic nasal splits for post-surgical recovery of rhinoplasty. In follow-up, it was found that there was a statistically significant difference (*p* < 0.05) in ecchymosis and edema on some of the days after surgery. They were able to conclude that their method produced significantly better results [55].

Zhu et al. used 3DP models to plan ear reconstruction surgery. Forty patients were split into a control group using a 2D template and one with used the models to plan the surgery. The results showed that subjectively the outcomes of the reconstructed auricle were more satisfactory in appearance (*p* = 0.005), shape (*p* = 0.026), size (*p* = 0.001), and similarity (*p* = 0.001) when the 3D model was used [56].

Living donor liver transplantation is becoming increasingly popular, during which it is crucial to optimize donor’s safety at donor hepatectomy. Zein et al. produced 3DP models of donor and recipient donors to identify vascular and biliary anatomical variation to facilitate surgical planning [57]. The models were highly accurate in volumetric analysis with a mean dimensional error of < 4mm. The models were helpful in characterizing biliary anatomy in one case in which intraoperative cholangiography was extremely difficult and time-consuming.

There is a paucity of realistic simulators to train complex laparoscopic procedures such as choledochal cyst excision and reconstruction. Burdall et al. developed a 3DP simulator through an SLA printer, which was received 5.6 ± 1.71 points for tactile realism, 6.2 ± 1.35 points for complexity, and 7.36 ± 1.57 points for usefulness (out of 10 points) by 10 delegates at a surgical workshop [58]. These reports demonstrate how complex procedures, which might be difficult to residents to learn through theatre experience, can be taught and practiced through 3DP simulators.

A significant limitation of 3DP is the expensive nature of incorporating its use in clinical practice. A 2017 report has shown the feasibility of creating a cost-effective model for complex laparoscopic resection of colorectal liver metastasis. The model was created for under USD 150 compared to previous reports, with models costing over USD 1000 [59]. The authors report that their transparent liver model preserved relevant anatomical and tumor characteristics allowing an uneventful R0 resection of liver metastasis laparoscopically. Similar cost-effective models for training laparoscopic splenectomy were also reported, suggesting that with increasing 3DP technology expertise, it is possible to reduce associated costs [60].

Printed models can significantly improve outcomes for patients thanks to the ability to better understand the operating space with three dimensions instead of two. Allowing surgeons to have physical models directly improves their ability to account for issues that might occur during the surgery and helps them better understand where caution needs to be taken.

### 2.6. Orthopedic Surgery

During preoperative planning, the DICOM images of the patients are used to create 3D-printed 1:1 scale representations of the operation site. These to-scale prints are then used by the surgeon to familiarize themselves with the site and perform instrumentation such as contouring [61]. Multiple small-scale trials suggest that operation time, blood loss, and application of fluoroscopy were able to be significantly reduced thanks to pre-operative planning using 3D-printed templates of the affected site [61,62,63].

During an interventional study (*n* = 48), Chen et al. examined the usefulness of 3D-printed 1:1 scale prints with regard to complex fractures of the distal radius and how these models would impact the patients understanding of their clinical situation. They observed that the group for which the 3D-printed models were utilized for pre-operational planning saw a significant decrease in all primary outcome measures: the mean operating time was significantly shorter in the 3D model group than in the routine group (66.5 ± 5.3 min vs. 75.4 ± 6.0 min, *p* < 0.001), blood loss volume was significantly decreased in the 3D model group over the routine group (41.1 ± 7.5 mL vs. 54.2 ± 7.9 mL, *p* < 0.05), and the frequency of fluoroscopy was also significantly lower in the 3Dp model group (5.6 ± 1.6 times vs. 4.4 ± 1.4 times, *p* = 0.011) [63].

When taking a look at the secondary outcome measures, the 3D printing technique is non-inferior. Here, the Gartland–Werley scores, radiological evaluation, and the range of motion of the wrist were examined. In all these cases, there was no significant difference noticed. Additionally, a questionnaire was filled out by the surgeons and by the patients. The scores for the questions “how much do you know about your fracture situation”, “how much do you know about your surgical plan” showed a significant difference from the 3D group to the routine group (*p* = 0.001); this was also reflected in the results from the questionnaire for the surgeons where the question “usefulness of the 3D prototype for communication with patients” was the highest-rated question (9.1 ± 0.8 points). However, it is important to note that despite the objective benefits of the 3D printing method, the majority (22) of surgeons answered “No” to the question “Would you use 3D printing models to treat a complex fracture again” whereas the minority (8) answered “Yes” to this question. Unfortunately, Chen et al. did not ask follow-up questions to the surgeons concerning the motivation for their answers; therefore, the reason for the discrepancy between the objective and subjective beneficial outcomes and the surgeons’ overall assessment remains unclear [63].

For patient-specific instrumentation, the 3D rendering of the affected site is used to construct a guiding template to assist the operator in reaching a more desirable outcome during the operation by assisting in guiding the instruments of the surgeon [64,65,66].

In a randomized controlled trial (*n* = 34), Wu et al. compared the use of an individualized 3D-printed template for Lateral Ankle Ligament Reconstruction for the treatment of Chronic Lateral Ankle Instability (CLAI) with the conventional use of a semitendinosus tendon autograft [64]. The DICOM data from the patients’ ankles were used to create the guide templates and a model of the ankle joint. After pre-operative planning, the guide templates were sterilized using plasma sterilization to enable safe use during the procedure. Wu et al. found that while the outcomes for both patient groups were satisfactory, the operation time (51.9 ± 3.6 min vs. 72.4 ± 12.6 min, *p* < 0.01) and the number of radiation exposures by fluoroscopy were significantly decreased in the template group (1.3 ± 0.6 times vs. 6.6 ± 1.7 times, *p* < 0.01) and therefore concluded that the new technique is more advantageous for the treatment of CLAI. It is worth mentioning that the other metrics used to compare the two groups with regard to the outcome showed no significant difference.

Case-specific implants are 3D-printed structures that are directly implanted instead of the autogenous tissue into the patient. Not unlike traditional implants, which have been used in orthopedic surgery for decades, these 3D-printed implants are manufactured in titanium using a powder-based Electron Beam Melting (EBM) 3D printer [65,67,68,69]. In addition, there are currently efforts underway to further the research in the use of artificial bone [70,71] and soft tissue [71,72,73]. However, a detailed discussion of this topic is beyond the scope of this paper. 

### 2.7. Otorhinolaryngology (Ear, Nose and Throat Surgery)

Particular interest in 3D printing has been observed in otosurgery [74,75,76,77]. Three-dimensional models of the temporal bones allow designing an operation for each patient individually. It is especially true in clinical cases of patients with developmental defects in the temporal bones. The average duration of the exercise ranged from 23.03 s to 62.77 s. Statistically significant differences were between the first and last time to finish training using the dominant hand (*p* < 0.05), and the mean completion time for younger and older residents (*p* < 0.05) suggest the usefulness of the 3D model of the temporal bone for training purposes [76]. On the printed model created based on CT of the patient’s temporal bones, the surgeon can plan operations and practice its separate stages, which improves the quality of treatment. In addition, 3D models of the external auditory canal and the tympanic membrane enabled students and younger residents in otolaryngology to perform tympanic membrane paracentesis and insert the ventilation drainages to the tympanic cavity before real-life procedures [78]. Frendo et al., in their study, observed a shortening of the learning curve among otosurgeons who used 3D models of the temporal bones in the education process of cochlear implant surgery. The learning curves were very individual but with constant improvement over 18 procedures. The insertion efficiency of the cochlear implant electrode on the 3D-printed temporal bone improved by 21% (*p* < 0.001) [74].

The virtual simulation also proved helpful before rhinoplasty. Gordon et al. conducted the study based on fifteen patients’ 3D-printed surgical templates. The final position of the nasal tip was on average 0.8 ± 0.7 mm from simulated projection and 0.3 ± 0.2 mm from simulated rotation. The authors concluded that the simulation of the stages of surgery and their possible consequences might assist the surgeon in making intraoperative decisions [79]. Virtual models are also useful in simulating airflow, and this can be used to assess in silico pre-procedurally and optimize the impact of planned procedures [80]. 

Functional endoscopic sinus surgery presents unique challenges for the surgeon due to its complex and variable anatomy and the risk of serious complications. The endoscopic sinus surgery training was facilitated by using 3D-printed simulators [81,82,83]. The multi-material and multi-color 3D printing technology create a realistic 3D model to simulate complex anatomical structures. In the Zheng et al. study, the multi-material and multi-color model was superior to the mono-material models in surgical training for exposing the vidian nerve, anatomical teaching, and preoperative planning (*p* < 0.05) [77].

Deonarain et al. created and validated a synthetic simulator for teaching tracheostomy and reconstruction of the larynx and trachea. Experts rated the face and content validity of the 3D model an overall median of 4 and 5, respectively, using a five-point Likert scale. There was no difference in scores between the synthetic model and the live porcine model for any surgery steps. The authors stated that the developed 3D model is of high quality, is not inferior to the pig model, and can teach students and younger residents [84].

The COVID-19 pandemic required, in a short time, the training of a large number of medical personnel to take a swab from the nasopharynx of patients. Therefore, Sananes et al. developed a 3D-printed simulator of nasopharyngeal smear sampling to improve the habits of swab collection [85].

### 2.8. Head and Neck Surgery

Surgical resection remains the mainstay of curative treatment for malignant and benign locally invasive tumors of the head and neck. Clinically or radiologically evident skeletal involvement in advanced disease necessitates the segmental removal of involved bone in isolation (e.g., mandibular ameloblastoma) or en-bloc with a soft-tissue neoplasm (e.g., locally-advanced squamous cell carcinoma of the oral cavity). Proper oncologic resection requires clearance of adequate surgical margins, which determines the size of the resultant bony defect and contributes to the attendant structural deformity and functional deficit.

By disrupting the structural integrity of the upper aero-digestive tract, head and neck surgery often impairs its normal functions such as breathing, alimentation, and speech. Subsequent restitution of these roles relies on prolonged rehabilitation and the use of prostheses. Effective reconstruction is aimed at reducing the deleterious effects of head and neck resection on patients’ aero-digestive function, facial cosmesis, and overall quality of life. Computer-aided design (CAD) coupled with 3D printing, i.e., computer-aided manufacturing (CAM), have revolutionized head and neck reconstruction.

Segmental mandibular defects were reconstructed with a composite free fibula flap pedicled off of the peroneal artery since 1989 [86]. Among other features, the fibula is selected for its ample pedicle, consistent linear shape, and adequate length, which permit segmentation via several osteotomies and the creation of relatively complex constructs customized to match the original mandibular projection and facial contour [87]. 

Historically, this customization process was performed by the surgeon free-hand during the reconstructive surgery itself, following the division of the vascular pedicle (conventional free-hand mandible reconstruction, CFMR), as described by Pellini et al. [88]. Due to the complex morphology of the mandible, the manual CFMR is challenging, while its expediency and accuracy are highly dependent on the surgeon’s skill and experience.

In contrast, the recent advent of computer-assisted planning and 3D printing has enabled greater precision and speed of the reconstructive process by moving much of the planning phase from the operating table to a pre-operative planning and fabrication lab (computer-aided mandible reconstruction, CAMR), as described by Hirsch et al. [89] and Succo et al. [90].

**Figure 8:**
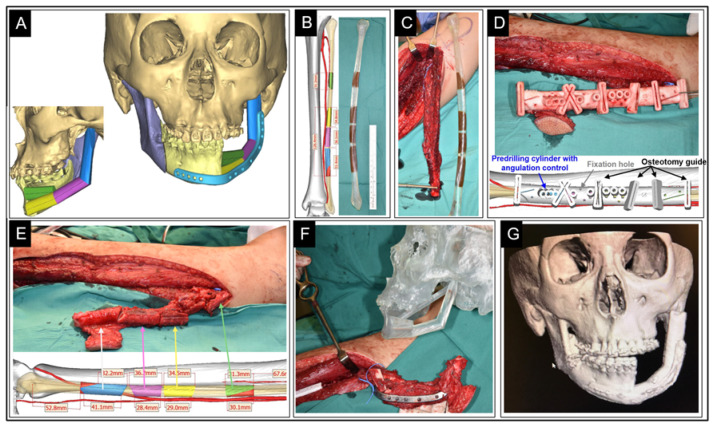
Three-dimensional printing in free fibula flap mandible reconstruction following odontogenic tumor resection: (**A**) Computer (CAD) plan of a reconstructed mandible; (**B**) Computer model and 3D print of fibula with planned construct segments marked out; (**C**) 3D-printed model next to actual patient’s fibula in vivo; (**D**) 3D-printed jig with osteotomy (cutting) guides on fibula; (**E**) segmented fibula in vivo and computer model with corresponding segments labeled; (**F**) assembled construct fixed with pre-bent metal reconstruction plate, prior to division of vascular pedicle; (**G**) CT image of patient three months following surgery. (Images courtesy of Dr. Chew Khong-Yik, Department of Plastic Surgery, Singapore General Hospital, Singapore).

In the author’s institution’s CAMR workflow, high-resolution computed tomography (CT) DICOM files are used to create a 3D computer model of the patient’s mandible, upon which the surgical oncologist digitally superimposes the planned resection planes. The reconstructive surgeon then uses another computer model derived from an angiographic CT scan of the patient’s leg to plan the free fibula construct. A technician then fabricates individualized life-size stereolithographic prints of the two models, as well as of custom snap-on cutting jigs, which are used to guide subsequent osteotomies and drill holes for fixation plates in vivo. The 3D-printed models are also used to precisely pre-bend the metal reconstruction plates and to plan for osteointegrated dental implants (Figure 8).

Preoperative planning, which consists of computer-aided design (CAD) and computer-aided manufacturing (CAM), contributes to an improvement in the reconstructive process and better outcomes. In a retrospective review of 57 patients undergoing FFF reconstruction for cancers involving the mandible, CAMR significantly shortened the total operative time compared to CFMR from 707 min to 534 min (*p* < 0.0003) [91]. In a series comparing 11 CFMR and 18 CAMR cases, Culie et al. demonstrated a reduction in ischemic time by half in favor of CAMR (75 min vs. 150 min, *p* <0.001), with greater time savings with the increased complexity of the reconstruction [92]. More recently, Blanc et al. compared operative and ischemic times between 14 CAMR and 16 CFMR patients, with the former yielding a gain of 88 min (*p* < 0.001) and 36 min (*p* = 0.04), respectively [93]. Finally, a meta-analysis comparing CFMR with CAMR concluded that 3D planning conferred a significantly shortened ischemic time by 35 min (−34.81, 95% CI, −40.07 to −29.55, *p* < 0.01), as well as improved reconstructive and total operative times [94].

In addition to operative and ischemic time gains attributable to CAD/CAM, CAMR has enabled more complex surgeries [95] with a high degree of accuracy in mandibular reconstruction compared to preoperative virtual models [93,96], with a mean error as low as 1 mm (range 0.4–2.46 mm) reported by Tarsitano et al. [97]. A systematic review of subjective cosmetic (e.g., facial appearance, aesthetic/social activity satisfaction) and functional (e.g., regular diet, intelligible speech) outcomes by Powecharoen et al. likewise favors CAMR over CFMR [94].

Although CAMR is intuitively associated with a greater up-front cost compared with that of CFMR, this pre-operative investment can be offset by savings in the operating time and superior outcomes. While the monetary cost of CAMR is likely to vary significantly across countries and healthcare systems, in a 2016 study of consecutive 9 CAMR and 11 CFMR performed over 2 years in one Swiss institution, Zweifel et al. demonstrated that the additional cost of virtual CAD-CAM to be nearly significantly by savings in operating theatre time alone (USD 5,098.00 vs. USD 1,231.50 with a pre-bent reconstruction plate and USD 6,980.00 vs. USD 3,113.50 for a milled one) [98]. Nonetheless, further studies to assess the economic viability of CAD-CAM mandibular reconstruction in various healthcare settings are needed.

The downsides of CAMR include the extended preparatory time required for CAD-CAM compared with CFMR (on the order of seven to ten days in the author’s institution), access to a specialized 3D-planning and -printing laboratory, as well as the availability of trained personnel.

In summary, mandibular reconstruction with a vascularized free fibular flap remains the gold standard, while the CAMR/CAD-CAM method brought about a paradigm shift towards personalized and precision surgery, with the incorporation of 3D modeling technology representing the current state-of-the-art. A similar approach was adapted to the reconstruction of other bony head and neck defects, including maxillary, orbital floor, base-of-skull, and cranial, and was demonstrated in related disciplines of otorhinolaryngology, oral, maxillofacial, and neurosurgery. Lastly, once fabricated, the 3D models continue to serve as valuable teaching aids for surgical trainees as well as communication aids in pre-operative counseling of future patients.

### 2.9. Neurosurgery

**Figure 9:**
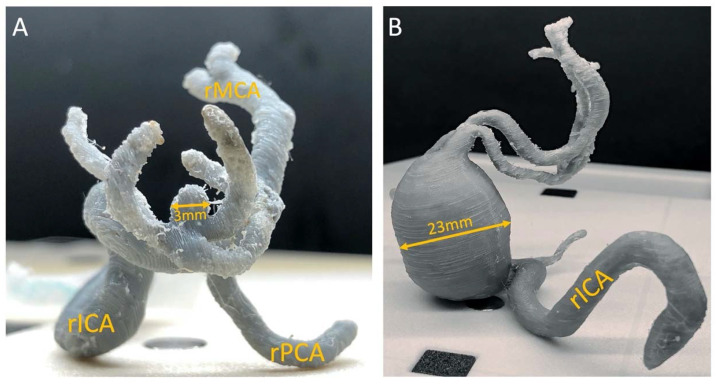
Three-dimensional-printed models of brain aneurisms (arrows) based on digital subtraction angiography: (**A**) An Anterior Communicating Artery / Right Anterior Cerebral Artery A2 (AcomA/ACA A2 dex) aneurysm. (**B**) A giant aneurysm of the right internal carotid artery (rICA). rPCA – right Posterior Cerebral Artery, rMCA – right Middle Cerebral Artery.

Intracranial aneurysms, vascular malformations, and complicated or difficult to locate vessels associated with brain tumors are just some of the pathologies that affect neurosurgical patients. Three-dimensional printing made it possible not only to create individual vascular models for each patient (Figure 9A,B) but also made it possible to prepare for surgery through training models for surgeons more accurately and to better explore the hemodynamics of these phenomena through scientific studies [99]. Currently, models are made with the greatest attention to detail so that the material fully reflects not only the shape of anatomical structures but also the tissues of which individual structures are made. As a result of such a multidisciplinary approach, we obtained a fully dimensional model of the patient’s head, taking into account other structures such as the brain, meninges, skull bones, sinuses, and intracranial vascularization [100]. Unfortunately, the necessity to approach each case on a case-by-case basis is a serious limitation in the efficiency of this practice due to its time- and resource intensity. Another limitation to the dissemination of the method is still a lack of widespread understanding of the 3DP technology.

One of the first proofs of the principle of facilitating “in-house” 3D printing for preprocedural planning in pediatric neurosurgery is a pilot study of four cases from Boston in 2015 [101]. Three of the patients had arteriovenous malformations (AVMs), and one had a vein of Galen malformation (VOGM). The fidelity of the models was cross-checked with the intraprocedural imagery, and concordance was found to be 98%. The use of 3D models conferred a 30 min reduction in operative time (12%) compared to matched controls.

Just two years later, Weinstock et al., using the help of Hollywood special effects technicians, created a modular training head that mimicked a 14-year-old patient’s noncommunicating hydrocephalus. They conducted a validation of the Endoscopic third ventriculostomy (ETV) simulation model, using a 14-item questionnaire to assess the face and content validity. Five senior and eight junior neurosurgery residents and four neurosurgery fellows were enrolled; the mean score for the content validity questions (4.88 points) was statistically higher than the mean score for face validity (4.69 points, *p* = 0.03) [102]. The authors also present an assessment of the quality of the EVT models made using the Objective Structured Assessment of Technical Skills (OSATS) scale, taking into account seven parameters (respect for tissue, time and motion, instrument handling, knowledge of instruments, flow of operation, use of assistants, and knowledge of the specific procedure), graded from 1 to 5 points each. The practice of endoscopic third ventriculostomy resulted in significant improvement in the flow of operation (*p* = 0.0004), time and motion (*p* = 0.0004), and use of assistants (*p* < 0.0001) using the OSATS scale [102]. Fellows’ grades were significantly higher than residents, same as senior residents had significantly higher grades for time and motion and instrument handling. These differences are probably due to more advanced surgical skills and greater knowledge of respondents. Weighted kappa agreement statistics on the evaluation of the performance of the participants showed high agreement scores between raters [102].

Planning brain tumor resections require MRI or CT to visualize the boundary between the mass of the tumor and the surrounding structures as accurately as possible. Nevertheless, intraoperatively, even bright images may not be sufficient to assess the adjacent structures or the tumor vasculature itself. Three-dimensional printing allows one to create a patient-specific model of the tumor mass and its location in relation to the brain, vessels, and bones of the skull. Additionally, on the basis of functional MRI (fMRI), the model may take into account the areas of the eloquent cortex that should be avoided during resection, also based on Diffusion-tensor magnetic resonance (MR) imaging (DTI) and fiber tractography (FT), elements visualizing the direction and continuity of the nerve fibers can be presented [103]. Surgical treatment of skull base tumors is extremely demanding and requires careful planning. Fully volumetrically colored 3D prints allow for the provision of a surgical simulation model that incorporates a variety of complex anatomical structures, often embedded in one another [104]. As in the case of vascular pathologies, neurooncological models are improved with appropriate materials, exactly reflecting the tissue architecture of each structure. These accurate models are used in practice not only as part of the preparation of surgery but also as important simulators for improving surgical skills. Moreover, 3D printing is not only used for anatomical models but also a tool used in radiotherapy of brain tumors, such as the proton range compensator device [103].

Transpedicular screws are one of the most popular tools used in spine deformity correction surgeries. However, incorrect anatomy and pedicle structure make it difficult to insert the screws properly, as well as rotational deformities make it more difficult to set the appropriate angle of the pedicle screw. A new solution with the use of 3D printing to facilitate spinal deformity surgery was introduced. Based on the preoperative imaging, the engineering team designs drill templates customized to the individual patient’s bony surface anatomy and the specific required trajectory for optimal placement of the transpedicle screws. It was shown by Cecchinato et al. [105] that using patient-specific surgical guides allows for more precise screws placement (8.2% vs. 17.1%, *p* < 0.0022, misplaced screws in the 3DP guided vs. freehand group). Moreover, less time per screw (6 min 10 s vs. 9 min 11 s, *p* = 0.002) also meant less fluoroscopy shots (11.0 times vs. 47.5 times, *p* = 0.001) and less radiation: DAP 133.5 ± 59.6 cGycm2 vs. 473.3 ± 448.3 cGycm2, exposure time 9.38 ± 4.87 s vs. 28.34 ± 27.68 s and mean effective dose in 3DP A 0.23 mSv vs. 0.82 mSv in free-hand group, respectively. This is an alternative to classical methods based on intraoperative navigation and robotic systems, which are not always available [106]. Similar ex vivo virtual planning of spinal instrumentation was studied for the thoracic spine [107].

The educational aspect is extremely important in spine surgery. Despite the continuous development of the quality of spine imaging, studies on students show that with 3D images, it is much easier to diagnose spine fractures than in classic 2D imaging [3]. The limited number of cadaveric courses and the number of course participants anticipated by the organizers make the availability of this type of training very limited, and most residents are unable to afford such a luxury, apart from further training and consolidation of acquired knowledge. Therefore, the use of 3DP models to learn procedures such as cervical laminectomy is an important step in the methodology of educating young surgeons [108]. Still, additional research needs to be conducted to develop this method.

Other procedures that require training and simulation are endoscopic procedures such as ventriculostomy or transsphenoidal endoscopic pituitary surgeries [103]. In ventriculostomy simulations, the 3DP reusable models were enriched with cerebrospinal fluid with the flow maintained under the appropriate pressure [109].

### 2.10. Gynecology and Obstetrics

Many single case reports and short studies presented the implementation of ultrasound-, CT-, or MRI-based 3D multi-material printing to prepare an individual patient-matched medical model to optimize treatment strategy of different complex procedures comprising, for instance, multispecialty fetoscopic surgery [110], hands-on operative planning, and surgical training in various fields of gynecological surgery [111].

First and foremost, in the light of growing interest in 3D printing technology, particularly in the cardiovascular community, its use in obstetrics essentially increased over the last few years [112]. Due to the anatomical variability, the complexity of interventional/surgical repair is very high; thus, the modeling in the treatment strategy, particularly in congenital heart disease (CHD), warrants an individual approach in preoperative planning [112]. The modeling, generated via image segmentation/volume rendering processing from 3D echocardiography, computed tomography (CT), micro-CT, or cardiovascular magnetic resonance imaging (MRI) imaging [113], is fast and more accessible, affordable, and those of good quality increases the patient’s confidence about their condition [113]. In our era, the possibility to visualize small cardiac structures and to navigate into normal and pathological fetal hearts offered by micro-CT facilitates the interpretation of ultrasound. In some complex cases of fetal cardiac anomalies, a novel way of using a physical 3D model for intracardiac flow pattern estimation was used to alter the virtual 3D models to increase wall size and add barbed connectors to create a flow model for 4D flow MRI [114]. This approach is valuable by providing an in vitro individual model to study alternations in flow dynamics in challenging heart defects as well as for parents to understand the complexity of cardiac anomaly [114]. Even a spatiotemporal image correlation (STIC) technology to display the spatial structure of the fetal heart seen on 2D screens does not provide as much information as a physical 3D-printed model [115].

In order to guide practitioners in the field, new approaches, including guidelines [116,117,118], and training delivery were proposed comprising, for instance, congress courses providing an overview of the principles of 3D printing, workflow from image acquisition, software packages, solutions available for image processing, and available materials. Currently organized courses provided, for instance, a description of potential cardiovascular applications of 3D technology, including decision-making, device testing, and first-in-human real-life cases based on CT or MRI data using different applications/printing options focusing on the technical features. Interestingly, the survey administered after a short teaching session focused on cardiovascular 3D printing during international congress indicated that in-house 3D printing service should be gradually implemented alongside multimodal imaging into clinical practice [113]. This approach pre-identified potential obstacles and errors, optimized surgical instrumentation, maximized intraoperative efficiency, and substantially decreased operative time [110]. 

Thus, 3D printing may be used in many different areas of operative planning and surgical training among obstetricians and gynecologists, including the planning of a complicated cesarean delivery [119], surgical planning for placenta accreta, treatment planning for cervical and endometrial cancer, and surgery for Mullerian anomalies and other benign gynecologic surgeries [111,119,120,121]. For instance, the knowledge of individual anatomy and pathology of the uterine cavity may essentially assist with fetal delivery from the myomatous uterus [119]. In these cases, an optimal uterine incision reduces the risk of myomectomy or hysterectomy at the time of uterine closure by decreasing the hemorrhage risk or fetal malpresentation [119]. 

Patient-specific 3D models derived from 2D MRI images provide a more accurate anatomical view, i.e., assessment of anatomical volumes, orientation relative to adjacent structures, gives a better understanding of surgical anatomy, potentially better outcomes, decreased operative time, blood loss, and incision length [63,111,122,123]. Interestingly, fields of interest may be printed in a bright opaque colored material to maximize visualization of pathologic tissue [111].

In cases of symptomatic uterine fibroids, common benign tumors affecting young women suffering from symptoms, i.e., heavy menstrual bleeding, pelvic pain, or infertility, printed models facilitate the assessment of the relationship of the uterine fibroids with surrounding anatomical structures, i.e., endometrium and myometrium, minimizing the procedure time, blood loss, and preserving the integrity of the endometrial lining [111]. In cases of endometriosis, preoperative planning is critical due to the recommendation of complete surgical excision within one procedure and the risk of unexpected complexity of the disease, especially in deep endometriosis and laparoscopic approach. Physical models improve the preparation for highly complex cases, including severe endometriosis by visualizing the extent of endometriotic nodules, suspicious for intraoperative complications [111].

In summary, over the last few years in the field of obstetrics and gynecology, we witnessed an increase in stepwise implementation of physical visualization by 3D printing modeling to help trainees in education or facilitate practitioners in preoperative assessment of disease complexity. They are useful to explain to patients the unique aspects of their surgeries, including surgical steps and surgical challenges. Undoubtedly, the implementation of 3D modeling in obstetrics and gynecological procedures will transform the landscape of treatment strategies in the future.

### 2.11. Urology

Most of the research on 3D printing as a tool for preoperative planning in urology refers to renal and prostatic cancer surgeries. One of the main challenges of these procedures is avoiding positive surgical margins, which has a key impact on the patient’s prognosis. 

Maddox et al. demonstrated fewer positive margins (0% vs. 7.4%) when using patient-specific models to simulate robot-assisted partial nephrectomies preoperatively [124]. Komai et al. proposed R.E.N.A.L. nephrometry 3D models printed in such a way that the tumor and the margin can be removed, allowing surgeons to verify both pre- and post-tumor resection kidney status and evaluate the margins of resection. All 10 patients included in the study had negative surgical margins, and the resected 3DP tumor nearly matched the surgical results. Furthermore, navigation using the 3D-printed kidney significantly shortened the duration of intraoperative ultrasound (mean 3.3 min) with a 3D model compared to without the 3D model (6.3 min, *p* = 0.021) [125]. Shin et al. used translucent resin prostate models with lesions and neurovascular bundles printed on the basis of magnetic resonance imaging (MRI). All five patients, despite being challenging high-risk cases (pT2c [*n* = 1], pT3a [*n* = 2], and pT3b [*n* = 2]) had negative surgical margins after robot-assisted radical prostatectomy [124,126,127,128,129]. 

Moreover, 3D printing has also been applied for preoperative planning of complex urolithiasis cases. Li et al. facilitated comprehensive planning for percutaneous nephrolithotomy (PCNL) with 3D models of a pelvicalyceal system with renal stones and percutaneous renal access route. Three-dimensional models were used successfully in all 15 patients. The one-stage stone-free rate was 93.3%. The authors concluded that 3D models help minimize the risks of PCNL and achieve higher one-stage stone-free rates, yet no comparative analysis was performed [130].

**Figure 10:**
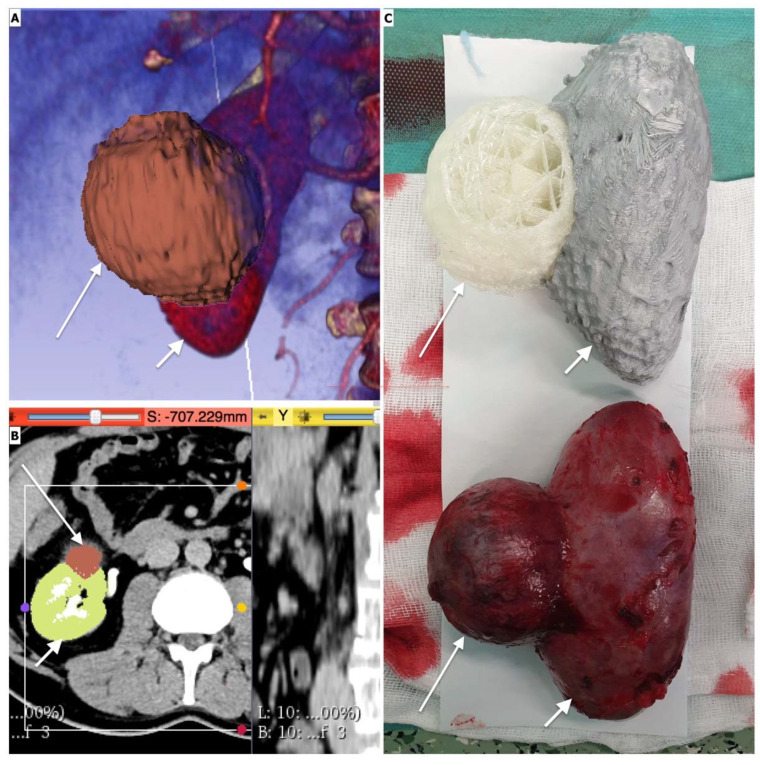
Three-dimensional prints of kidney anatomy; (**A**) Virtual 3D reconstruction based on contrast-CT scan of the kidney (short arrow) with the tumor (long arrow). (**B**) Contrast-CT scan of the kidney (short arrow) with the tumor (long arrow). (**C**) Top—3D-printed model of the kidney (short arrow) with the tumor printed with a separate color (long arrow); Bottom—resected kidney (short arrow) with the tumor (long arrow). Nephrectomy was necessary due to the tumor penetrating into the renal calyx. The 3D print model corresponded to the actual size of the kidney and the tumor.

Creating 3D-printed, personalized training models seems to be an ideal application of this technology. Numerous authors reported the use of models of organs for surgical training. Most of them investigated the usefulness of 3D prints of kidney anatomy with tumors and surrounding vessels prior to nephrectomy or NSS (Figure 10).

Von Rundstedt et al. used preoperative imaging of 10 patients and 3D printing to create renal silicone models to practice patient-specific robot-assisted laparoscopic partial nephrectomy. There was no significant difference in volumes between the original 3D reconstruction from the CT scan, the resected pre-surgical model, and the resected tumor specimen (*p* = 0.98). There was also no difference in resection times between the patient-specific pre-surgical models and the actual surgery (6 min 58 s vs. 8 min 22 s, *p* = 0.162). Not only were these models complete tools for the increase in surgical skills, but they also provided an objective evaluation of efficiency and progress before hands-on exposure [131].

An interesting application of 3D printing technology for improving prostate targeted biopsy was presented by Wang et al.; a 3D print assisting cognitive fusion targeted biopsy avoided the missed diagnosis of two cases (20%), effectively improving the positive biopsy rate [132]. Translucent 3DP models of prostates were also found to be very helpful when attempting to biopsy patients with a high risk of positive margins. Shin et al. used these see-through models to find the best approach, helping avoid the capsule and neurovascular bundle in five patients. They were able to achieve negative margins in all five patients [127].

Other applications included creating 3D-printed models of bladder and calyces printed with a translucent, acrylonitrile butadiene styrene-like plastic material being optimal for flexible ureteroscopy training. Fifteen residents underwent a flexible ureteroscopy course based on the printed training models. The mean post-course task completion times (15.76 vs. 9.37 min, *p* = 0.001) and overall performance scores (19.20 vs. 25.25, *p* = 0.007) were significantly better than at baseline [133]. 

An ideal application for 3D printing would be the creation of patient-specific ureteral stents, as due to the reflux mechanism, standard stents often cause dysuric symptoms. The in vitro research conducted by Park et al. showed that creating anti-reflux ureteral stents with polymeric flap valve using 3D printing is feasible and such stents effectively prevented backward flow with minimal reduction in forward flow. Backward flow rates were decreased by 8.3 times (uncoated valve) and 4.0 times (parylene C coated valve) at an applied pressure of 50 cm H_2_O, respectively, compared with the intact DJ stent [134]. On the other hand, 3D-printed surgical clips proposed by Canvasser et al. proved inefficacious as they broke, failed to close, and leaked more than commercially available alternatives. The fracture rate during closure of the printed clips was 54% (*n* = 27), while none of the Hem-o-lok clips broke (0%, *p* < 0.0001) [135]. Similarly, 3D-printed trocars proposed by Junco et al. formed larger superficial defect areas compared to two conventional trocars (29.41 mm^2^ vs. 18.06 mm^2^ vs. 17.22 mm^2^, *p* < 0.001) [136].

High hopes for the development of 3DP are also placed in bioprinting. The major aim is to print biological tissues that could be safely used in patients. In urology, tissue-engineered kidneys usable for transplantations seem to be the main direction of bioprinting development. Moreover, research on bioprinting of the urethra for patients with hypospadias or urethral stricture is still ongoing. However, bioprinted individual renal structures or small organoids are the greatest achievements so far. Ali et al. bioprinted renal constructs that exhibited the structural and functional characteristics of the native renal tissue [137]. The authors designed a photo-cross-linkable kidney extracellular matrix-derived bio-ink (KdECMMA) that could provide a kidney-specific microenvironment for renal tissue bioprinting. Despite demonstrating the potential of such tissue-specific bio-ink, further developments, in particular regarding the bioprinting of vascular networks and refining the tissue scaffolds, is the biggest challenge. 

A significant hurdle in bioprinting the urethra and other organs is obtaining the multi-layered structure of a tissue, mimicking the real organ. Thus, such tissue must be strong enough to be used during reconstructive surgeries, such as urethroplasty, as the ultimate aim of culturing tissues is to enable the replacement of damaged or absent organs [138].

Three-dimensional printing has already found its place in urology, as shown in the recent detailed reviews [139,140]. Additionally, there are several urological procedures in which presenting the nature of the disease and its complexity to the patient is particularly useful. The use of 3D-printed models during patients’ preoperative counseling has been reported for nephrectomy, nephron-sparing surgery (NSS), radical prostatectomy, and percutaneous nephrolithotomy (PCNL). Personalized organ models improved the patients’ understanding of the essence of the pathology, planned surgery, and surgical risks [125,126,141].

### 2.12. Emergency Medicine and Anesthesiology

The rapid development of 3D printing has created a new learning and teaching tool for anesthesiology training and pre-procedural planning. These applications are mostly related to simulation models for airway management, bronchoscopy training, front-of-neck access, spinal/neuraxial procedures, vascular and intraosseous access, and pericardiocentesis.

In recent years we could observe innovative applications of 3D printing in the field of cardiovascular emergencies and vascular access management. For instance, Pang et al. demonstrated the use of a 3D printer to build a pelvis model with a vascular access system that was integrated with commercially available cardiopulmonary resuscitation manikin for multi-disciplinary extracorporeal cardiopulmonary resuscitation training. While there is a range of commercially available manikins for ECMO simulation purposes, Pang’s model is unique because it enables simulation along a continuum from basic (first responders, paramedics) to advanced (emergency and critical care physicians), and finally, extra-corporeal life support are contained within a single medical unit [142]. As another example, Baribeau et al. constructed a low-cost (~ USD 200), high-fidelity, ultrasound-guided training phantom that offered the opportunity to practice pericardiocentesis without the time pressure of an emergency situation. During a 5-day perioperative transthoracic echocardiography course, four fellows rating (1–5 scale) the satisfaction on the training method (3.75 ± 1.5 points), accustoming of performance the procedures (3.5 ± 1.0 points), the realism in palpation for the xiphoid process (3.5 ± 1.3 points), the effectiveness of echo-guidance (3.25 ± 1.5 points), the depth of the pericardium and corresponding needle entry into the pericardium (3.5 ± 1.3 points and 3.25 ± 1.5 points) [143]. Similarly, using easy-to-purchase 3D printing components, Lord et al. produced a durable pericardiocentesis chest model for less than USD 110 (material costs) [144]. As for vascular access, Tan et al. compared a 3D-printed ultrasound-guided peripheral intravenous catheter (US-PIVC) phantom and five times more costly (USD 120 vs. USD 549) commercial model in terms of time to placing a US-PIVC by 21 emergency medicine physicians and found that there was no significant difference in mean time PIVC placement and realism between the two groups [145]. In another study, Engelbrecht et al. found the 3D-printed trainer to be a practical tool for practicing intraosseous techniques among rural medicine residents, rural emergency medicine physicians, and nurses [146].

Recently, more advanced and realistic models for bronchoscopy and surgical airway training have been developed. Maier et al. established a set of 3D-printed pediatric (infant, toddler, adolescent) static and dynamic airway models for bronchoscopy and foreign body removal. The models were evaluated by three pediatric pulmonary attendings, and according to their judgment, the dynamic airway model (consisting of a flexible tree connected to a pump) provides the most realistic representation of a pediatric airway during the respiratory cycle. Currently, the models are used as part of the training for pediatric pulmonary fellows and otolaryngology residents at Children Hospital of Philadelphia [147]. Leong et al., in a commentary article for Pediatric Pulmonology, described a high-fidelity infant 3D model with realistic haptic qualities used for experience with bronchoalveolar lavage and clearing of secretions, as well as other invasive bronchoscopic procedures [148]. Ho et al. presented a similar approach in adults, whereby 3D airway models were used for stimulation training of bronchoscopy in the setting of airway pathologies, such as a tumor obstructing the right main bronchus and a goiter causing external tracheal compression [149]. In terms of surgical airways, Kei et al. created a realistic cricothyrotomy simulator with skin, bleeding, and flash of air to alleviate some of the barriers surrounding an emergent cricothyrotomy. The model was designed to provide the user with an affordable, easy to replicate, and realistic experience. Moreover, the authors provided an online video, a list of supplies, a 3D trachea STL file, and step-by-step building instructions for recreating the trainer [150]. Dziedzic et al. successfully used a 3D-printed model to assess a tracheal wall collapse due to tracheobronchomalacia. The model assisted in preplanning the procedure, and despite the difficult nature of the pathology, they were able to widen the trachea and main bronchi successfully [151].

Epidural analgesia is believed to be the most difficult technique to learn for a trainee. In order to address this issue, Han et al. designed a do-it-yourself 3D-printed thoracic model for epidural needle placement, which 10 anesthesia experts and 15 novices evaluated as a very good and helpful learning tool. The model consisted of five thoracic vertebrae and discs, the ligamentum flavum, and surrounding soft tissue. The production cost of the model was around USD 40 and was significantly lower than that of commercially available products. The fabrication time of the 3D-printed phantom was 14 h, and the hands-on build time of 5 h. In order to contribute experience and implement ideas of collaborative effort in the wider community, the authors released 3D printer files and an assembly manual through an open-source website [152]. Similarly, Odom et al. developed a 3D lumbar spine model for training palpate and ultrasound-guided lumbar punctures. The model was utilized by residents, advanced practice providers, and medical students who found it easy to use and realistic during palpation and US-guided stimulation training [153].

Although the three-dimensional (3D) printing technology has been successful in the field of pre-operative planning in surgical interventions, there are not many studies in the field of anesthesia. One single-center study was conducted by Park et al. and demonstrated better results of a printed 3D airway model (60%) in the prediction of the correct size of endotracheal tubes for endotracheal intubation than the age-based formula (26%) in pediatric patients with congenital heart disease [154]. There is also the potential of using a 3D model of a specific patient for preprocedural training and planning. For example, Shaylor et al. described the use of three-dimensional printing and virtual reality to produce a personalized airway plan in a 7.5-year-old child scheduled for right upper lobectomy due to lung metastasis from an Ewing’s sarcoma. The team used data from CT scans to develop the three-dimensional airway printing and converted it into a virtual reality bronchoscopy. Following the planning and rehearsal processes, the team was able to perform successful isolation and ventilation of the patient’s left lung [155]. More single-patient case studies were reported in the literature and focused on the management of the difficult pediatric airway in Jarcho–Levin syndrome [156] and pre-anesthesia planning in the patient after total laryngectomy [157].

### 2.13. Radiotherapy

Radiation oncology is a fundamental component of cancer treatment. Of all cancer patients, 50% require radiotherapy during the course of their disease [158]. For safety reasons, delivering radiation requires high precision in patient setup and immobilization. The need for personalization resulted in interest in adopting 3D printing for radiotherapy. However, up to now, this technique is still not widely used in everyday practice. According to PubMed, only 327 articles about 3D printing in radiation oncology were published (search term: “3d print radiotherapy”), and only one commercially available software can convert DICOM-RT data into ready-to-print digital 3D models (Adaptive Medical Technologies Inc.). 

**Figure 11:**
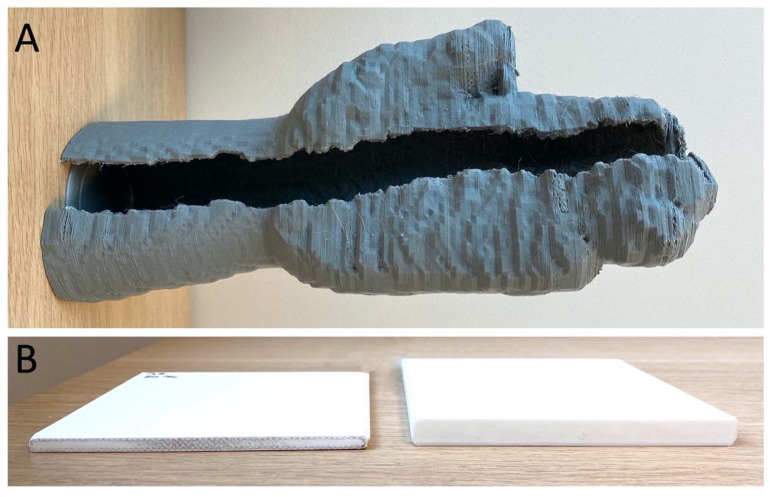
Applications of 3DP in radiotherapy. (**A**) An individually created hand bolus for total body irradiation before an allogenic stem cell transplant. (**B**) Boluses of different thicknesses (0.5 and 1 cm) with different infill percentages (60% and 20%) changing their physical properties.

One of the most commonly 3D-printed accessories for radiotherapy is boluses [159]. Boluses are artificial materials with physical properties equivalent to human tissues. For each patient, the bolus is shaped to fit a body surface to compensate for missing tissues or to increase the dose on the skin surface (Figure 11A). According to the literature, 3D-printed boluses are not worse than commercial ones. Dyer et al. conducted research comparing density, clarity, and net bolus effect between a planar commercial bolus and a 3D-printed bolus for head and neck radiotherapy. They used Clear Stratasys TangoPlus 3D (Stratasys Ltd., Eden Prairie, MN) for printing. The 3D-printed boluses improved radiotherapy plan conformity (median Conformity Index for 3D-printed bolus—0.993, for commercial bolus—0.977), reduced air gap volumes in irregular superficial areas (such as the ear, orbit, and nose) from median 17 cc to 7 cc, and provided excellent dose coverage [160]. Another indication for implementing 3D printing into radiotherapy is the production of anthropomorphic phantoms. Phantoms are used to investigate an organ or a whole-body effective dose as well as for the verification of delivery of therapeutic radiation doses. In an ideal world, phantoms should be a perfect copy of an irradiated patient, which was impossible before the era of the 3D print. Moreover, 3D printing allows for creating very complex structures, e.g., a prostate gland with an intraprostatic lesion, seminal vesicles, urethra, ejaculatory duct, neurovascular bundles, rectal wall, and penile bulb—all generated from a series of contrast-enhanced magnetic resonance images [161]. 

Material selection and printing parameters affect bolus final physical properties [162,163], but this fact can be used to advantage (Figure 11B). Another advantage of 3D printing is the possibility of combining different materials with different physical properties in one phantom, as was described by Hazelaar et al., They used gypsum and nylon, different dose absorbing materials, with different Hounsfield Units (HU) to develop a chest phantom closely resembling a real patient [164].

3D printing was also adopted in brachytherapy—a branch of radiotherapy in which a radioactive source is placed directly near the tumor or tumor bed. In feasibility studies, printed applicators for cervical, skin, or prostate cancers not only met the necessary requirements in quality control tests [165] but also seemed to be better than commercial ones in terms of dose distribution [166,167]. In Logar et al.’s study, 3D-printed gynecological applicators improved one of the most important parameters in brachytherapy—mean D90; the mean dose encompasses 90% of the clinical target volume, increasing it from 14.1 ± 5.4 Gy to 22.0 ± 2.5 Gy and from 7.1 Gy to 16.2 Gy for cervical/recurrent endometrial and vaginal cancer, respectively [166]. Arenas et al. conducted a financial study comparing the costs of 3D printing of skin cancer applicators (molds) with the usual custom handmade mold protocol. They found that the new technology reduced time and costs spent on making molds by 34% and 50%, respectively [168]. The 3D-printed templates for radioactive source implementation also seem superior to conventional planning procedures. By obtaining optimal radioactive source distribution, they optimized dose distribution and reduced the implementation time (standard mean difference, SMD = −0.93) [169]. 

Three-dimensional printing seems to be useful in training programs for interstitial brachytherapy. As was shown by Chiu et al., printing low-cost, reusable phantoms are a cost-effective platform to gain procedural skills in brachytherapy. The material cost for each phantom was approximated at USD 23.98, and the time needed to prepare a complete phantom was 1.5–2 h [170]. However, in studies describing the development of prostate brachytherapy training phantoms, 3D printing was used only for printing inverse molds, not organs, which were created from silicone, gelatin, agar, or polyvinyl chloride [170,171]. Campelo et al. showed that it is possible to print entirely and directly the whole phantom in 3D without using molds. They used a Stratasys J750 PolyJet printer using an Agilus and Vero Clear photopolymers with different Shore A values to print gynecological phantom. The cost of materials to manufacture the kit was approximately USD 631 [172]. Up to now, there are no studies describing practical aspects of training radiation oncology residents using 3D-printed phantoms, and further research on this topic is expected.

However, there are some limitations in implementing 3D printing into radiotherapy. First of all, every material used in printing must be examined as to how it reacts to ionizing radiation. As was shown by Biltekin et al., infill-percentage, infill-pattern, and printing direction significantly changed the dosimetric and physical properties of the 3D-printed boluses [165]. Another study showed that 3D-printed accessories, even when printed from the same material roll and on the same 3D printer, have large spreads in HU and density, which may be crucial for radiotherapy planning. The biggest differences were seen while using PLA and NinjaFlex (differences in HU up to 121 and 178, respectively); the smallest were for ABS and Cheetah (30 HU for both). According to the authors of the study, the properties of each 3D-printed accessory should be individually evaluated before using it in clinical practice [163]. Another obstacle in adopting 3D printing into everyday practice in radiation oncology facilities is the lack of software for DICOM-RT conversion into ready-to-print STL files. Open-source programs do not support this format; that is why most researchers use homemade software, such as those developed and shared by Nowak [173].

## 3. Discussion

Until recently, medical 3D printing may have been considered a gadget or a fad. However, it has proven to be a disruptive technology and is progressively accepted in clinical practice. As such, 3DP represents a step towards personalized medicine—a seemingly novel concept with roots in the very foundations of medicine. While, for some, the utility of 3DP remains limited, many find it useful in selected applications or in their most challenging cases. However, others have already incorporated 3DP into their standard workflows for specific indications. Understandably, as a novel technology, medical 3D printing has as many limitations and pitfalls as it promises. With very limited, or in certain specialties (e.g., cardiovascular medicine) virtually absent quality data relating to outcomes, assessment of true benefit to the patient is difficult. Hence, broad recommendations pertaining to 3DP on a clinical guideline level are currently unavailable. On the other hand, studies are emerging (and are cited above), which provide evidence that medical 3D printing is clearly a valuable adjunct to complex interventions and that the technology is here to stay. Table 1. provides a brief summary of the aforementioned applications. Supplementary Table S1. breaks down the discussed literature to greater detail. The reader should keep in mind that with the illustrative rather than exhaustive focus of the article, blank cells do not necessarily represent a lack of a particular application.

### 3.1. Investment and Operational Costs

Running an in-house 3D printing lab is costly, and outsourcing can cut the costs only in specific situations. The high upfront expenditures are related to capital investment in 3D printing equipment, whereas high maintenance costs are due to remuneration for the required specialized personnel. Nonetheless, 3D printing has the potential to offer substantial operational cost savings by shortening procedure times, increasing success rates, and resulting in fewer clinical complications. Moreover, increasingly more automated software, possibly augmented by artificial intelligence and computer vision (another disruptive technology), would further limit the time as well as manpower required to perform image segmentation and digital model processing. A robust cost-effectiveness analysis is currently needed to enable decisions with regards to the cost-effective adoption of 3DP in specific clinical scenarios and to inform possible models of reimbursement.

### 3.2. Print Fidelity 

The accuracy of 3D prints has always been a concern. The topic was well summarized in the recent review by Chae et al. [174]. The evidence from multiple studies shows that 3DP based on MDCT have acceptable accuracy for clinical application, with a mean difference from original anatomical specimens of less than 1–2 mm (1%–2%) across all printing techniques (SLA, BJT, PJT, FFF, SLS). There is variability across different printing technologies, but most of the error is being introduced during the image segmentation stage [174]. It is also worth considering the variability across different material types (esp. rigid vs. elastic), model type (solid vs. hollow), and model orientation at a printer bed even within the same printer [175], although it is minor (sub-millimeter) and relatively insignificant for most clinical applications. Therefore, it can be concluded that significant print inaccuracies are likely a result of user error. It is thus critical that inter-operator variability be assessed as part of the quality assurance process.

### 3.3. Radiation Exposure

Based on the literature, most of the 3D-printed models made today are derived from muti-detector computed tomography (CT). This is considered the modality of choice for most of the clinical scenarios owing to its higher 3D special resolution as compared to MRI and other imaging modalities. Although contrasted CT acquisition is associated with exposure to ionizing radiation and an iodinated contrast medium, the necessary images are often required as per the routine clinical workup. Hence, in many clinical scenarios, the utilization of 3DP technology does not increase harmful patient exposure. Nonetheless, efforts are made to apply radiation-less alternatives and to develop new image processing algorithms. One such recent example is the so-called synthetic CT (sCT) derived from post-processed MRI images, which is in no way inferior to standard CT. The bone models printed off sCT have been shown to have excellent agreement with ground truth based on distance mapping of the surface, and surgical cutting guide placement error (both x–y–z translation and x–y–z rotation) was also shown to be no different when compared between CT and sCT [176].

### 3.4. Mimicking Tissues’ Mechanical Properties

Novel models used for simulation and training are expected to truthfully mimic tissue properties such as flexibility, elasticity, breakage, etc. The range of investigated [177] as well as already commercially available materials for 3D printing is expanding, but there is still no good experimental data on which one to choose for a given application. Probably the biggest hurdle is we do not exactly know the mechanical properties of living tissues—neither healthy nor diseased. Hence, the development of next-generation 3D-printed models, which are accurate not only in terms of the anatomical fidelity but also in mimicking living tissue properties will rely not only on new material and print techniques, but also on the exact characterization of mechanical properties on the living tissues.

### 3.5. Bioprinting

One of the major anticipated advances expected from the ongoing progress in 3DP technology is in the bioprinting of custom individualized grafts, with the capability to manufacture patient-specific organs on demand representing the ultimate goal of the field of 3DP. Currently, bioprinting technology is in its nascency, and only relatively simple prints of bone, cartilage, and skin have seen successful grafting [178]

Bioactive composite scaffolds are being developed to assist bone regeneration using a variety of materials such as polymers, hydrogels, metals, ceramics, and bio-glasses in order to find the best material with which living tissue will bind [71,179,180]. Alternatively, a hydrogel medium mixed with various cell types can be used as bio-inks. Chea et al. used bio-inks in their process to create functional knee menisci [73]. Despite early promise, the current process of bioprinting is limited by the challenges of using multiple bio-inks with the aim of creating functional organs with several cell types and suitable vascularization. However, looking at the pace of technological development in recent decades, bioprinting mays have a revolutionary impact on surgical innovation and become a practicable tool in the near future.

### 3.6. Drug Delivery

Another potential application of 3DP is in the delivery of custom pharmaceutical formulations and dosages. The bioavailability of a drug differs from person to person depending on the weight, age, renal function, and other physiologic and metabolic parameters of the patient. Custom drug manufacturing enables the control of dosage and delivery methods [181]. Drugs can also be incorporated in the manufacturing process to impregnate implants or devices for direct and prolonged drug application [182]. Tablet printing gives one precise control over the release profile of a drug formulation by varying tablet infill and geometry [183]. On-demand customization of dosage and delivery is the future of personalized patient care tailored to each patient’s needs, rather than relying on mass-produced generic therapy.

### 3.7. Ethical and Legal Considerations

Finally, ethical and legal issues arise related to quality assurance, safety, and medicolegal accountability. Three-dimensional printing is a complex, time-consuming process involving multiple specialized actors. First in the chain is the patient themselves, having to cooperate during image acquisition. Second, the radiologist and the radiology technician must properly plan and carry out the imaging. This is dependent on excellent knowledge of the technology at hand and the clinical question, which the images are intended to answer in each specific clinical case. Next, there are clinicians and 3DP lab technicians who need to cooperate closely to generate an accurate model focused on the region of interest inclusive of relevant surrounding anatomy, which has the demanded visual and tactile properties of transparency, flexibility, multicolor palette, and varied stiffness, all dependent on the specific needs. Finally, there is careful inspection of the model, possibly simulation of the upcoming procedure, and making clinical judgments and decisions based on the model. This clearly demonstrates that medical 3D printing is a multi-step team effort involving multiple actors with distinct and critical responsibilities [184].

This complex interplay blurs the lines of accountability and sets the scene for the following ethical and legal considerations. Firstly, with so many actors, the process must be precisely standardized at every step, and the tools used validated and certified. The processing of every model must be documented to facilitate troubleshooting and investigation in the event of a model failure (a broad and yet not well-defined event). Secondly, the 3D-printed model can be classified as merely a form of medical documentation, an alternative way to represent already available imaging when used solely as a visual aid for inspection (not unlike CT volumetric reconstructions). Conversely, a model can also be considered a medical device when it is used interactively during procedure simulation or intervention (rehearsal, implant fitting and sizing, surgical guide, etc.). Because conventional medical devices are produced and quality-checked centrally, the certification process and responsibility for the final product rests with the manufacturer. In contrast, the disseminated nature of the 3DP process precludes such a conventional regulatory framework. Thus, only the tools (software, hardware) and the process itself can be certified, whereas the responsibility to adhere to the manufacturing procedure falls on the healthcare provider, and specifically on each actor involved in the 3DP process [184,185]. Although it is hard to find published documentation of a 3D-printed model having misled clinical decisions, it is not difficult to imagine such a scenario.

Moreover, the high upfront and maintenance costs involved in high-quality medical 3D printing raise questions on whether 3DP is poised to further expand healthcare inequity vis-a-vis less affluent societies [186].

## 4. Conclusions

Already, 3D printing is gaining acceptance as a technology capable of aiding education and training, procedure planning and rehearsal, as well manufacturing of custom medical tools and implants. In doing so, it is expected to find applications across more specialties and medical centers worldwide. Multiple examples of the use of 3D printing in medicine and a few studies supporting the clinical gains in 3D printing-aided patient-specific treatment mentioned above convince us that this disruptive technology is to stay in medicine for good. New techniques both in image acquisition processing as well as in printing technology are emerging. Our experience shows that there is a great interest in this new field, and introducing 3D printing at the level of undergraduate medical education helps not only to promote the technology but also to raise awareness of its limitations and future directions [187]. 

In conclusion, in the ever-expanding world of personalized medicine, where medical decisions and interventions are tailored to each individual patient, the contribution of 3DP will result in customized pre-operative planning, individualized simulated surgeries, custom patient-specific implants, and bio-printed organs/structures. It is evident that for patients with complex and rare conditions, preprocedural planning and simulation aided by 3DP can yield valuable benefits. In the domains of teaching, device development, procedures, and materials, 3DP can improve accuracy, safety, and efficiency by its unique ability to rapidly create prototypes using low-cost materials that are tailored to the individual. There has been growing recognition that the traditional teaching method of “see one, do one, teach one” is no longer adequate, with a move towards a “see one, do many with stimulation, do one” approach [188]. The relatively low-cost, custom 3D-printed models enabling realistic simulation are particularly complementary to this shift in training methodology. As a result, 3DP provides a safe environment to help increase the physician’s comfort with the procedure without exposing patients to unnecessary risk of harm associated with a procedural learning curve. However, before 3D printing can be widely deployed and embraced by the medical community, the current state-of-the-art calls for standardization of the tools and protocols used at every step of the process to optimize accuracy and minimalize inter-operator variability. To ensure the best possible model fidelity for use in the various clinical applications, careful choice of the methods, tools, and materials is key. Quality research, preferably multicenter randomized controlled trials, is necessary to characterize the benefits of this technology better, still novel in the field of medicine.

## Figures and Tables

**Table 1:** Summary of 3D printing applications. Numbers correspond to citations.

	Preprocedural Planning	Procedure Simulation	Professional Education
Pediatric Cardiac Surgery	[6,7,9]		[5,8]
Pediatric Interventional Cardiology	[11,12,13]	[10,12]	
Cardiac Structural Interventions	[14,15,16,20,21,22,23,27,28,29,31,32,33]	[14,17,18,19,30]	[26,27,28,29]
Pediatric Surgery	[37,38,39,40,41]	[34,35]	
General Surgery	[44,45,46,50,51,52,53,56,57]	[47,49,54]	[48,58,59,60]
Orthopedic Surgery	[62,63,64,65,66,122,123]	[61,66,122,123]	
Otorhinolaryngology	[77,79,80]		[74,75,76,77,78,81,82,83,84,85]
Head and Neck Surgery	[87,88,89,90,91,92,93,94,95,96,97,98]	[91,92,93,94,95,96,97,98]	[88]
Neurosurgery	[101,103,104,105,106,107]	[100]	[100,119,124,125,126,127,128,129,131,132,151]
Gynecology and Obstetrics	[110,111,112,113,114,115,120]	[119]	
Urology	[127,130]	[124,125,126,127,128,129,131,132]	[131,133]
Emergency Medicine and Anesthesiology	[151,154,155,156,157,158]	[151]	[142,143,144,145,146,147,148,149,150,152,153]
Radiotherapy	[160,162,163,164,166,168]		[166,168,170,171,172]

## Data Availability

No new data were created or analyzed in this study. Data sharing is not applicable to this article.

## Supplementary Materials

The following supporting information can be downloaded at: https://www.mdpi.com/article/10.3390/ijerph19063331/s1. Data of the relevant studies that are referenced in this paper are compiled in a supplementary table to parse this large set of data more easily. Table S1. Summary of the discussed 3D printing publications.
