# Supplementary material for: The Role of 3D Printing in Planning Complex Medical Procedures and Training of Medical Professionals—Cross-Sectional Multispecialty Review
Source: Int J Environ Res Public Health. 2022 Mar 11;19(6):3331. doi: 10.3390/ijerph19063331 (PMC8953417; doi:10.3390/ijerph19063331)
Supplement: Supplementary file 1 [file ijerph-19-03331-s001.zip › ijerph-1582441-supplementary.pdf]

Table S1. Summary of the discussed 3D printing publications.

Citation	Author	Year	Field	Anatomy/Pathology	Type of Intervention	Motive
5	Scanlan el al.	2018	Pediatric Cardiac Surgery	Atrioventricular valves	Valvular repair	Specialized Training
6	Valverde et al.	2017	Pediatric Cardiac Surgery	Congenital heart defects	Better understand unique patient pathology	Visualize Pathology
7	Deng et al.	2021	Pediatric Cardiac Surgery	Ventricular septal defect	Better understand unique patient pathology	Visualize Pathology, Educate Patients
8	Costello et al.	2015	Pediatric Cardiac Surgery	Ventricular septal defect	Better understand variations in the pathology	Specialized Training
9	Tiwari et al.	2021	Pediatric Cardiac Surgery	Congenital heart defects	Best route of intervention	Procedure Planning
10	Valverde et al.	2015	Pediatric Interventional Cardiology	Transverse aortic arch hypoplasia	Planning endovascular stenting	Procedure Simulation
11	Phillips et al.	2016	Pediatric Interventional Cardiology	Tetralogy of Fallot	Transcatheter pulmonary valve replacement	Procedure Planning
12	Jalal et al.	2018	Pediatric Interventional Cardiology	Atrioventricular septal defect will left ventricular outflow tract obstruction	Hybrid melody valve implantation	Procedure Planning, Procedure Simulation
13	Bhatla et al.	2017	Pediatric Interventional Cardiology	Ventricular septal defect	Right atriotomy with a tricuspid valve approach	Procedure Planning
14	Sabiniewicz et al.	2018	Cardiac Structural Interventions	Adults with congenital heart disease	Transcatheter Pulmonary Valve Replacement	Procedure Planning, Procedure Simulation
15	Biernacka et al.	2018	Cardiac Structural Interventions	Adults with congenital heart disease		Procedure Planning
16	Schievano et al.	2007	Cardiac Structural Interventions	Adults with congenital heart disease		Procedure Planning
17	Ripley et al.	2016	Cardiac Structural Interventions	Aortic Valve pathology	Transcatheter aortic valve replacement	Procedure Simulation
18	Zelis et al.	2020	Cardiac Structural Interventions	Aortic Valve pathology	Transcatheter aortic valve implantation	Procedure Simulation
19	Qian et al.	2017	Cardiac Structural Interventions	Aortic Valve pathology	Paravalvular leaks in transcatheter aortic valve implantation	Procedure Simulation
20	Levin et al.	2020	Cardiac Structural Interventions	Structural valve deteriorations	Valve in valve procedures	Procedure Planning
21	Little et al.	2016	Cardiac Structural Interventions	Mitral valve pathology	Percutaneous mitral interventions	Procedure Planning
22	Wang et. al.	2016	Cardiac Structural Interventions	Mitral valve pathology	Transcatheter mitral valve replacement procedure	Procedure Planning
23	Izzo et al.	2016	Cardiac Structural Interventions	Mitral Valve	Transcatheter Native Mitral Valve Replacement	Procedure Planning
24	Holmes et al.	2014	Cardiac Structural Interventions	Left atrial appendage		
25	January et al.	2019	Cardiac Structural Interventions	Heart	Arrythmia	
26	Wang et al.	2016	Cardiac Structural Interventions	Stroke with atrial fibrillation	Percutaneous left atrial appendage closure	Specialized Training
27	Pellegrino et al.	2016	Cardiac Structural Interventions	Left atrial appendage	Transseptal puncture site and device sizing	Procedure Planning, Specialized Training
28	Otton et al.	2015	Cardiac Structural Interventions	Left atrial appendage	Transseptal puncture site and device sizing	Procedure Planning, Specialized Training
29	Hell et al.	2015	Cardiac Structural Interventions	Left atrial appendage	Transseptal puncture site and device sizing	Procedure Planning, Specialized Training

30	Li et al.	2017	Cardiac Structural Interventions	Left atrial appendage		Procedure Simulation
31	Sabiniewicz et al.	2020	Cardiac Structural Interventions	Aortic arch aneurysm		Procedure Planning
32	Cruz-Gonzalez et al.	2019	Cardiac Structural Interventions	Valve pathology	Transcatheter paravalvular leak closure	Procedure Planning
33	ElGuindy et al.	2020	Cardiac Structural Interventions	Valve pathology	Transcatheter paravalvular leak closure	Procedure Planning
34	Bellia-Munzon et al.	2020	Pediatric Surgery	Pectus excavatum	Nuss procedure	Procedure Simulation, Custom Fit
35	Huang et al.	2019	Pediatric Surgery	Pectus excavatum	Nuss procedure	Procedure Simulation, Custom Fit
36	Deng et al.	2020	Pediatric Surgery	Pectus excavatum	Vacuum bells	Custom Device
37	Villarreal et al.	2020	Pediatric Surgery	Conjoined twin	Conjoined twin separation	Procedure Planning
38	Prayer et al.	2019	Pediatric Surgery	Congenital diaphragmatic hernias	Of patch placement	Procedure Planning
39	Sánchez-Sánchez et al.	2018	Pediatric Surgery	Tumor	Oncological surgery	Procedure Planning
40	Beltrami et al.	2021	Pediatric Surgery	Musculoskeletal Tumor	Surgical resection	Procedure Planning, Custom Implant
41	Vanesa et al.	2020	Pediatric Surgery	Mandibular hypoplasia	Mandibular distraction	Procedure Planning, Custom Implant
42	Morrison et al.	2015	Pediatric Surgery	Tracheobronchomalacia	Splints	Custom Implant, Bioresorbable Splints
43	Pietrabissa et al.	2020	General Surgery	Abdomen		
44	Pankaj et al.	2019	General Surgery	Kidney	Transplant	Procedure Planning
45	Chandak et al.	2017	General Surgery	Kidney	Transplant	Procedure Planning
46	Stefania et al.	2017	General Surgery	Kidney	Transplant	Procedure Planning
47	Rui et al.	2018	General Surgery	Biliary tree	Choledochoscopic Examination	Procedure Simulation, Custom Device
48	Hasan et al.	2016	General Surgery	Pelvis	Percutaneous Nephrolithotripsy Surgery	Specialized Training
49	Shibata et al.	2017	General Surgery	Visceral Aneurysm	Endovascular Embolization	Procedure Simulation
51	Marconi et al.	2016	General Surgery	Pancreatic cancer		Procedure Planning
52	Vater Fang et al.	2016	General Surgery	Pancreatic cancer or cancer around the ampulla of Vater	Surgical resection	Procedure Planning
53	Bauermeister et al.	2016	General Surgery	Pathologies of the skull	Various procedures	Procedure Planning
54	Richard et al.	2019	General Surgery	Laryngotracheal stenosis	surgical correction	Procedure Simulation
55	Erdogan et al.	2021	General Surgery	Nasal malformation	External nasal splints	Custom Implant
56	Zhu et al.	2016	General Surgery	Ear malformation	Ear reconstruction surgery	Procedure Planning
57	Zein et al.	2013	General Surgery	Liver failure	Liver transplantation	Procedure Planning
58	Burdall et al.	2016	General Surgery	Choledochal cyst	Choledochal cyst excision and reconstruction	Specialized Training
59	Witowski et al.	2017	General Surgery	Colorectal liver metastasis	Laparoscopic resection	Specialized Training
60	Pietrabissa et al.	2016	General Surgery	Colorectal liver metastasis	Laparoscopic resection	Specialized Training

61	Huang et al.	2020	Orthopedic Surgery	Acetabular joint	fracture	Procedure Simulation
62	Kong et al.	2020	Orthopedic Surgery	Intra-Articular Distal Radius	fracture	Procedure Planning
63	Chen et al.	2019	Orthopedic Surgery	Radius fracture	Complex fractures of the distal radius	Procedure Planning, Educate Patients
64	Wu et al.	2020	Orthopedic Surgery	Chronic Lateral Ankle Instability	Lateral Ankle Ligament Reconstruction	Procedure Planning
65	Park et al.	2021	Orthopedic Surgery	Pelvic tumor		Procedure Planning
66	Gu et al.	2020	Orthopedic Surgery	Knee	Arthroplasty	Procedure Planning, Procedure Simulation
67	Harrysson et al.	2008	Orthopedic Surgery		Implant	
68	Mazzoli et al.	2009	Orthopedic Surgery	Cranium	Implant	
69	Kantaros et al.	2021	Orthopedic Surgery		Scaffolds	
70	Cheng et al.	2021	Orthopedic Surgery		Bone Substitute	
71	Larson et al.	2020	Orthopedic Surgery		Bioprinting	
72	Wang et al.	2021	Orthopedic Surgery		Implant	
73	Chae et al.	2021	Orthopedic Surgery		Implant	Bioink
74	Frendø et al.	2021	Otorhinolaryngology	Hearing loss	Cochlear implant surgery	Specialized Training
75	Frithioff et al.	2021	Otorhinolaryngology	Temporal bone		Specialized Training
76	Barber et al.	2016	Otorhinolaryngology	Temporal bone	Defect repair	Specialized Training
77	Zheng et al.	2019	Otorhinolaryngology	Nasal Sinus		Procedure Planning, Specialized Training
78	Ferreira et al.	2021	Otorhinolaryngology	External auditory canal and the tympanic membrane	Tympanic membrane paracentesis	Specialized Training, Student Training
79	Gordon et al.	2021	Otorhinolaryngology	Nasal malformation	Rhinoplasty	Procedure Planning
80	Tretiakow et al.	2020	Otorhinolaryngology	Nasal malformation	Simulating airflow	Procedure Planning
81	Moniripiri et al.	2021	Otorhinolaryngology	Nasal Sinus	Endoscopic sinus surgery	Specialized Training
82	Haemmerli et al.	2021	Otorhinolaryngology	Nasal Sinus	Endoscopic sinus surgery	Specialized Training
83	Valtonen et al.	2020	Otorhinolaryngology	Nasal Sinus	Endoscopic sinus surgery	Specialized Training
84	Deonarain et al.	2021	Otorhinolaryngology	Larynx and trachea	Tracheostomy and reconstruction	Specialized Training
85	Sananes et al.	2021	Otorhinolaryngology	Nasopharynx	Taking a swab	Specialized Training, Student Training
86	Hidalgo et al.	1989	Head & Neck Surgery	Mandible	Reconstruction with a composite free fibula flap	
87	Mavrogenis et al.	2019	Head & Neck Surgery	Segmental mandibular defects	Reconstruction with a composite free fibula flap	Procedure Planning
88	Pellini et al.	2012	Head & Neck Surgery	Mandibular pathology	Conventional free-hand mandible reconstruction	Procedure Planning, Specialized Training
89	Hirsch et al.	2009	Head & Neck Surgery	Mandibular pathology	Computer-aided mandible reconstruction	Procedure Planning
90	Succo et al.	2015	Head & Neck Surgery	Mandibular pathology	Computer-aided mandible reconstruction	Procedure Planning
91	Toto et al.	2015	Head & Neck Surgery	Mandibular pathology	Conventional free-hand mandible reconstruction, computer-aided mandible reconstruction	Procedure Planning, Procedure Simulation

92	Culié et al.	2016	Head & Neck Surgery	Mandibular pathology	Conventional free-hand mandible reconstruction, computer-aided mandible reconstruction	Procedure Planning, Procedure Simulation
93	Blanc et al.	2019	Head & Neck Surgery	Mandibular pathology	Conventional free-hand mandible reconstruction, computer-aided mandible reconstruction	Procedure Planning, Procedure Simulation
94	Powcharoen et al.	2019	Head & Neck Surgery	Mandibular pathology	Conventional free-hand mandible reconstruction, computer-aided mandible reconstruction	Procedure Planning, Procedure Simulation
95	Monaco et al.	2016	Head & Neck Surgery	Mandibular pathology	Conventional free-hand mandible reconstruction, computer-aided mandible reconstruction	Procedure Planning, Procedure Simulation
96	Wu et al.	2021	Head & Neck Surgery	Mandibular pathology	Conventional free-hand mandible reconstruction, computer-aided mandible reconstruction	Procedure Planning, Procedure Simulation
97	Tarsitano et al.	2018	Head & Neck Surgery	Mandibular pathology	Conventional free-hand mandible reconstruction, computer-aided mandible reconstruction	Procedure Planning, Procedure Simulation
98	Zweifel et al.	2015	Head & Neck Surgery	Mandibular pathology	Conventional free-hand mandible reconstruction, computer-aided mandible reconstruction	Procedure Planning, Procedure Simulation
99	Cogswell et al.	2020	Neurosurgery	Intracranial vasculature		
100	Nagassa et al.	2019	Neurosurgery	Cerebral artery aneurysm		Procedure Simulation
101	Weinstock et al.	2015	Neurosurgery	Arteriovenous malformations and vein of Galen malformation		Procedure Planning
102	Weinstock et al.	2017	Neurosurgery	Noncommunicating hydrocephalus	Endoscopic third ventriculostomy	Specialized Training
103	Randazzo et al.	2016	Neurosurgery	Brain tumor	Resection	Procedure Planning
104	Kosterhon et al.	2020	Neurosurgery	Skull base tumors	Resection	Procedure Planning
105	Cecchinato et al.	2019	Neurosurgery	Spine deformity	Spine deformity correction	Procedure Planning
106	Chen et al.	2019	Neurosurgery	Spine deformity	Spine deformity correction	Procedure Planning
107	Clifton et al.	2020	Neurosurgery	Spine deformity	Spine deformity correction	Procedure Planning
108	Weiss et al.	2019	Neurosurgery	Spine fractures	Cervical laminectomy	Specialized Training
109	Waran et al.	2015	Neurosurgery	Ventricles of the brain	Ventriculostomy or transsphenoidal endoscopic pituitary surgeries	Specialized Training

110	Miller et al.	2018	Gynecology and Obstetrics	Congenital heart disease	Fetoscopic surgery	Procedure Planning
111	Flaxman et al.	2021	Gynecology and Obstetrics	Symptomatic uterine fibroids, endometriosis	Resection	Procedure Planning
112	Sandrini et al.	2020	Gynecology and Obstetrics	Congenital heart disease		Procedure Planning
113	Biglino et al.	2019	Gynecology and Obstetrics	Congenital heart disease		Procedure Planning
114	Ruedinger et al.	2018	Gynecology and Obstetrics	Congenital heart disease		Procedure Planning
115	Huang et al.	2021	Gynecology and Obstetrics	Congenital heart disease		Procedure Planning
116	Abudayyeh et al.	2018	Cardiac Structural Interventions	Heart		
117	Mitsouras et al.	2015	Radiotherapy			
118	Otton et al.	2017	Cardiac Structural Interventions	Heart		
119	Mackey et al.	2019	Gynecology and Obstetrics	Delivery	Cesarean delivery	Specialized Training
120	Baek et al.	2016	Gynecology and Obstetrics	Cervical Cancer		Procedure Planning
121	Barsky et al.	2018	Gynecology and Obstetrics	Pelvis	Pessary	
122	Punyaratabandhu et al.	2018	Orthopedic Surgery	Cancer		Procedure Planning, Procedure Simulation
123	Corona et al.	2018	Orthopedic Surgery	Tibia	Post-traumatic correction	Procedure Planning, Procedure Simulation
124	Maddox et al.	2018	Urology	Renal cancer	Robot-assisted partial nephrectomies	Procedure Simulation
125	Komai et al.	2016	Urology	Renal cancer	Resection	Procedure Simulation
126	Porpiglia et al.	2018	Urology	Prostate cancer	Robot-assisted radical prostatectomy	Procedure Simulation
127	Shin et al.	2016	Urology	Prostate cancer	Robot-assisted radical prostatectomy	Procedure Simulation
128	Silberstein et al.	2014	Urology	Prostate cancer	Robot-assisted radical prostatectomy	Procedure Simulation
129	Jomoto et al.	2018	Urology	Prostate cancer	Robot-assisted radical prostatectomy	Procedure Simulation
130	Li et al.	2013	Urology	Renal stones	Percutaneous nephrolithotomy	Procedure Planning
131	von Rundstedt et al.	2017	Urology	Renal cancer	Robot-assisted laparoscopic partial nephrectomy	Procedure Simulation, Specialized Training
132	Wang et. al.	2015	Urology	Prostate cancer	Biopsy	Procedure Simulation
133	Blankstein et al.	2015	Urology	Bladder and kidney	Flexible ureteroscopy	Specialized Training
134	Park et al.	2015	Urology	Ureteral reflux	Ureteral stents	Custom Implant
135	Canvasser et al.	2017	Urology		Surgical clips	Custom Implant
136	Junco et al.	2015	Urology		Trocars	Custom Device
137	Ali et al.	2019	Urology	Kidney		Bioprinting
138	de Kemp et al.	2015	Urology	Urethra	Reconstruction	
139	Chen et al.	2020	Urology			
140	Cacciamani et al.	2019	Urology			
141	Atalay et al.	2017	Urology	Pelvis		
142	Pang et al.	2020	Emergency Medicine and Anesthesiology	Pelvis		Specialized Training, Student Training

143	Baribeau et al.	2020	Emergency Medicine and Anesthesiology	Thorax	Perioperative transthoracic echocardiography	Specialized Training
144	Lord et al.	2021	Emergency Medicine and Anesthesiology	Thorax	Pericardiocentesis	Specialized Training
145	Tan et al.	2021	Emergency Medicine and Anesthesiology		Ultrasound-guided peripheral intravenous catheterization	Specialized Training
146	Engelbrecht et al.	2020	Emergency Medicine and Anesthesiology		Intraosseous technique	Specialized Training, Student Training
147	Maier et al.	2021	Emergency Medicine and Anesthesiology	Airway	Bronchoscopy	Specialized Training
148	Leong et al.	2021	Emergency Medicine and Anesthesiology	Airway	Bronchoalveolar lavage and clearing of secretions	Specialized Training
149	Ho et al.	2019	Emergency Medicine and Anesthesiology	Airway	Bronchoscopy in the setting of airway pathology	Specialized Training
150	Kei et al.	2019	Emergency Medicine and Anesthesiology	Airway	Cricothyrotomy	Specialized Training, Student Training
151	Dziedzic et al.	2020	Emergency Medicine and Anesthesiology	Tracheobronchomalacia	Mesh and ring	Procedure Planning, Procedure Simulation
152	Han et al.	2020	Emergency Medicine and Anesthesiology	Lumbar spine	Epidural analgesia	Specialized Training, Student Training
153	Odom et al.	2019	Emergency Medicine and Anesthesiology	Lumbar spine	Palpate and ultrasound-guided lumbar punctures	Specialized Training, Student Training
154	Park et al.	2021	Emergency Medicine and Anesthesiology	Airway	Endotracheal intubation	Procedure Planning
155	Shaylor et al.	2020	Emergency Medicine and Anesthesiology	Airway	Ventilation	Procedure Planning
156	Olomu et al.	2020	Emergency Medicine and Anesthesiology	Airway	Ventilation	Procedure Planning
157	Han et al.	2016	Emergency Medicine and Anesthesiology	Airway	Ventilation	Procedure Planning
158	Atun et al.	2015	Emergency Medicine and Anesthesiology	Airway	Evaluation	Procedure Planning
159	Rooney et al.	2020	Radiotherapy	Cancer		
160	Dyer et al.	2020	Radiotherapy	Head and neck cancer	Bolus for radiotherapy	Procedure Planning, Custom Device
161	Lee et al.	2018	Radiotherapy	Prostate cancer		
162	Biltekin et al.	2021	Radiotherapy		Bolus for radiotherapy	Procedure Planning, Custom Device
163	Craft et al.	2018	Radiotherapy		Bolus for radiotherapy	Procedure Planning, Custom Device
164	Hazelaar et al.	2018	Radiotherapy	Thorax	Bolus for radiotherapy	Procedure Planning, Custom Device
165	Biltekin et al.	2020	Radiotherapy	Vagina	Brachytherapy	
166	Logar et al.	2019	Radiotherapy	Female reproductive cancer	Brachytherapy gynecological applicators	Procedure Planning, Specialized Training, Custom Device
167	Semeniuk et al.	2021	Radiotherapy		Brachytherapy	
168	Arenas et al.	2017	Radiotherapy	Skin	Skin cancer applicators	Procedure Planning, Specialized Training, Custom Device
169	Chen et al.	2021	Radiotherapy			
170	Chiu et al.	2020	Radiotherapy	Prostate	Interstitial brachytherapy	Specialized Training, Custom Device
171	Doyle et al.	2021	Radiotherapy	Prostate	Brachytherapy	Specialized Training
172	Campelo et al.	2020	Radiotherapy	Pelvis	Gynecological phantom	Specialized Training, Custom Device
